# Spatiotemporal Variations of Soil Reactive Nitrogen Oxide Fluxes across the Anthropogenic Landscape

**DOI:** 10.1021/acs.est.3c05849

**Published:** 2023-10-19

**Authors:** Megan L. Purchase, Gary D. Bending, Ryan M. Mushinski

**Affiliations:** School of Life Sciences, University of Warwick, Coventry CV4 7AL, United Kingdom

**Keywords:** soil emissions, reactive nitrogen oxides, land use, air quality, climate change, human impact

## Abstract

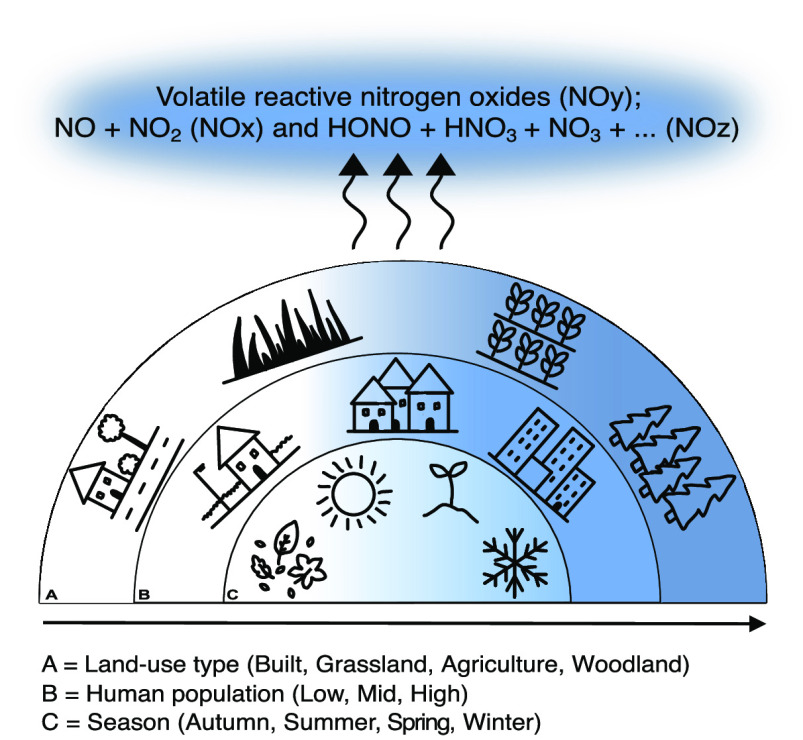

Volatile reactive nitrogen oxides (NO_*y*_) are significant atmospheric pollutants, including NO_*x*_ (nitric oxide [NO] + nitrogen dioxide [NO_2_]) and NO_*z*_ (nitrous acid [HONO] + nitric acid [HNO_3_] + nitrogen trioxide [NO_3_] + ...). NO_*y*_ species are products of nitrogen (N) cycle processes, particularly nitrification and denitrification. Biogenic sources, including soil, account for over 50% of natural NO_*y*_ emissions to the atmosphere, yet emissions from soils are generally not included in atmospheric models as a result of a lack of mechanistic data. This work is a unique investigation of NO_*y*_ fluxes on a landscape scale, taking a comprehensive set of land-use types, human influence, and seasonality into account to determine large-scale heterogeneity to provide a basis for future modeling and hypothesis generation. By coupling 16S rRNA amplicon sequencing and quantitative polymerase chain reaction, we have linked significant differences in functional potential and activity of nitrifying and denitrifying soil microbes to NO_*y*_ emissions from soils. Further, we have identified soils subject to increased N deposition that are less microbially active despite increased available N, potentially as a result of poor soil health from anthropogenic pollution. Structural equation modeling suggests human influence on soils to be a more significant effector of soil NO_*y*_ emissions than land-use type.

## Abstract 2

Emissions of reactive nitrogen oxides (NO_*y*_) from soils are currently understudied. This study reports changes in rates of nitrogen cycling processes and NO_*y*_ fluxes from soils between seasons, across a land-use continuum, and as a function of anthropogenic influence.

## 1 Introduction

**Figure 1:**
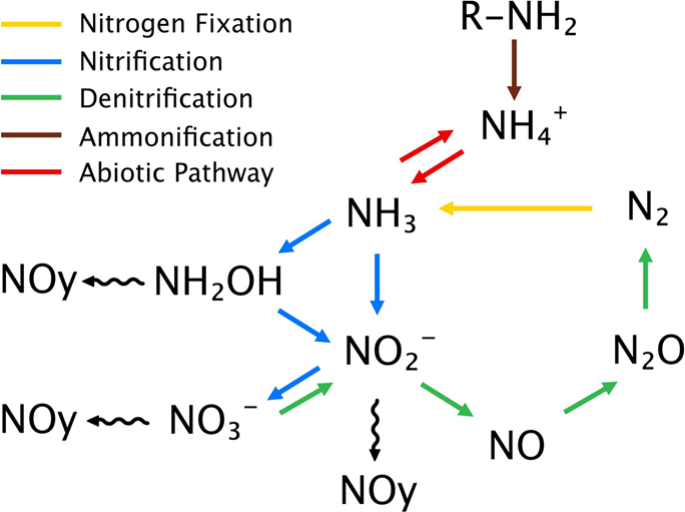
Overview of nitrogen cycle processes: nitrogen fixation, nitrification, denitrification, ammonification, and nitrogen fixation. Wavy lines indicate production of reactive nitrogen oxide gases (NO_*y*_).

Volatile reactive nitrogen species (NO_*y*_) are comprised of NO_*x*_ (nitric oxide [NO] + nitrogen dioxide [NO_2_]) and NO_*z*_ (nitrous acid [HONO] + nitric acid [HNO_3_] + nitrogen trioxide [NO_3_] + ...). NO_*y*_ is a pollutant that decreases air quality, contributes to global warming, and can negatively impact human health. Cycling and emissions of NO_*y*_ can be directly linked to nitrogen (N) cycling, primarily the processes of nitrification and denitrification (Figure 1). As a result of the profound effect of human activities on the global climate, the majority of research on NO_*y*_ focuses on emissions from anthropogenic sources. These include vehicle exhausts and byproducts of industry and agriculture as well as the effects of increasing temperatures resulting from climate change.^1−3^ In recent years, NO_*y*_ emissions directly from anthropogenic sources have declined, making emissions from soils increasingly relevant and necessitating quantification.^4,5^ Soil emissions of NO_*y*_ are primarily dependent upon microbial processes and interactions as well as other non-biogenic factors, but these mechanisms are currently not well-understood.

NO_*y*_ species can be emitted during the process of nitrification, and therefore, nitrification rates in soils can be directly related to NO_*y*_ emissions.^6^ Nitrification involves the oxidation of ammonia (NH_3_) to nitrite (NO_2_^–^) and nitrate (NO_3_^–^), accomplished in a two-step reaction involving ammonia- and nitrite-oxidizing microbes. The first step of nitrification is carried out by ammonia oxidizing archaeal (AOA) and bacterial (AOB) taxa, producing the enzyme ammonia monooxygenase (AMO). These taxa are included in bacterial orders Nitrosomonadales and Nitrosphaerales as well as archaeal order Cenarchaeales. AMO is coded for by genes *amoA*, *B*, and *C*, of which *amoA* is commonly used as a marker for taxonomic and functional analyses. The key enzyme of nitrite oxidation, NXR, is a specific marker for the second step of nitrification, which is a major biological source of NO_3_^–^ in the environment. More recently, it has been found that nitrification can be accomplished in one step by comammox (complete ammonia oxidation) taxa, primarily Nitrospirales. Nitrogen gas (N_2_) is formed from the anaerobic respiration of NO_2_^–^, NO, and nitrous oxide (N_2_O) in a process called denitrification. Denitrification leads to the release of nitrogen oxide gases from the soil to the atmosphere (Figure 1), of which N_2_O has gained the most attention because of its high global warming potential and contribution to ozone depletion.^7,8^ AOB taxa can also be involved in the denitrification process via nitrifier denitrification.^9^

Landscape fragmentation involves the separation of continuous habitats, which results in reduced areas of certain habitats and isolation of patches of land uses.^10^ Globally, ∼38% of land surface is classified as agriculture by the Food and Agriculture Organization (FAO) of the United Nations, and in England, ∼69% of land is classified as agriculture by the U.K. Government Department for Environment, Food & Rural Affairs.^11−13^ Despite numerous studies investigating NO_*y*_ emissions from this dominant land-use type, we still lack an understanding of how land-use change affects abiotic and biotic cycling of NO_*y*_ on a landscape scale.^1,7,14^ Accelerated conversion of land to residential and industrial areas is a function of human population growth and the associated demands for space in urban areas and resources. Soils found in these built environments are those that have been created or modified by humans as well as those altered by anthropogenically caused environmental changes, creating distinctive soil habitats. Urban soils are an increasingly important subject for study because more than 50% of the global population now live in urban areas.^15^ Urbanization leads to a land-use type gradient, with differences in soil reactive N fluxes expected to be observed in areas of greater human influence as a result of higher potential N inputs via deposition of N species from the atmosphere to the soil. Here, “human influence” refers to the overall impact of anthropogenic activities on soils through areas of higher human populations and human-driven changes in land uses. It has been reported that NO_*y*_ emissions from woodland soils are a function of the microbial community, soil physicochemical properties, and rates of N cycling processes. In particular, the activities of AOA and AOB as part of the nitrification pathway have been linked to production of NO_*y*_.^6,16^ Input of organic matter into soils differs between land-use types, determining the C/N ratios of dissolved organic matter (DOM) and, therefore, impacting the rates of N cycling processes.^17^ Woodland soils in particular are exposed to increased litter deposition, with the specific nature of the litter dependent upon the plant species present.^18^ As a result of decomposition of organic matter by heterotrophs, it is common for woodland soils to be relatively acidic compared to other land-use types, and excess H^+^ protons may have consequences for pathways that lead to NO_*y*_ emissions.^19^ Grass-dominated soils tend to contain higher concentrations of DOM than agricultural soils but lower than woodland soils, primarily as a result of the diversity of vegetation.^18^ Even more so than research on agricultural soils, reports on grass-dominated soils are focused on N_2_O emissions, and little attention is given to production of NO_*y*_ gases from this land-use type.^20^

In addition to higher levels of N deposition in urbanized areas, other biogeochemically active elements may be introduced to soils via anthropogenic means. Heavy metals and metalloids can naturally enter the environment through volcanic activity and weathering of minerals; however, particularly since the Industrial Revolution, anthropogenic activities have led to contamination of the natural environment by an excess of heavy metals and metalloids.^21,22^ Although heavy metals are required for some physiological processes, overaccumulation can have detrimental effects for the soil microbial community, including N cycling microbes, by altering the structure of nucleic acids and proteins and disrupting enzymatic activity.^22^ While some data exist to quantify the concentrations of heavy metals in soils, there is little consideration given to how increased or altered levels will affect tangential biogeochemical processes, including the N cycle, within the soil. The coupling of NO_*y*_ flux measurements to heavy metal concentrations and degree of urbanization may help constrain soil sourced NO_*y*_ in atmospheric models.

When potential differences in microbial diversity and activity between soils across landscapes are investigated, it is also pertinent to consider temporal effects. However, this temporal variable is often excluded from studies. Season-specific conditions (e.g., temperature, water content, etc.) can have a marked impact on microbial composition and activity, which can, in turn, affect N cycling processes.^23−25^ The optimal temperature for bacterial and fungal growth has been reported to be between 25 and 30 °C, and as temperatures increase or decrease beyond this range, growth rates decrease. However, bacterial growth is more inhibited by low temperatures than high temperatures.^23^ The soil moisture content often fluctuates in the environment. The optimal moisture content for bacterial activity differs between species, and aerobic bacteria in particular can be adversely affected by a high soil moisture content.^24^

In this work, we investigate four land-use types that represent the total land-use gradient of the U.K.: agricultural, woodland, grassland, and built. We take a comparative approach investigating land-use type and human influence to elucidate direct and indirect influences on NO_*y*_ emissions from soils through alterations to abiotic and biotic soil properties. We hypothesize that (1) land-use type affects reactive N fluxes through variation to soil physicochemical properties, (2) reactive N fluxes and the associated soil microbial community fluctuate with seasonal environmental variation, and (3) human influence on soils, including deposition of N species and heavy metals, affects reactive N fluxes.

## 2 Methods

### 2.1 Soil Sampling

Soil was sampled seasonally over 1 year in November 2021, February 2022, May 2022, and August 2022. Mean U.K. precipitation (mm) and temperature (°C) data for these months can be found in Table S2 of the Supporting Information. Soil was sampled from three locations with differing human populations: (1) low population, Wellesbourne, U.K., <1000; (2) mid-population, Warwick, U.K., 35 000–52 000; and (3) high population, Coventry, U.K., >400 000. Within these locations, samples were taken from four land-use types: agricultural, woodland, grassland, and built. Here, we define “built” soil as that located within 1 m of a human made structure, such as buildings and car parks (Figure S1 of the Supporting Information). There were three replicates of each land-use type within each location. Samples were taken in four seasons (spring, summer, autumn, and winter), giving a total of 132 total samples. At each sampling site, soil was sampled using a 7 cm diameter core to a depth of 5 cm. Three samples were taken at each site at 5 m intervals along a 15 m transect and pooled to produce one biological replicate. The 10 cm depth intact cores were also taken and used for continuous NO_*y*_ gas flux analysis. One core per land-use type was collected for each location for gas analysis, and samples were taken in three seasons (spring, summer, and winter), giving a total of 36 cores. Samples were transferred on ice packs to the laboratory and stored at 4 °C, and processing began within 7 days. All samples other than intact cores for gas analysis were then passed through a 2 mm sieve. Subsamples of each were taken and stored in Eppendorf tubes at −80 °C for DNA extraction. Soil texture and parent materials of the sample sites can be found in Table S1 of the Supporting Information.

### 2.2 Soil pH, Water Content, and Heavy Metal Quantification

Soil pH was determined using a pH meter on a solution of 10 g of soil, air-dried for 48 h, in 20 mL of 0.01 M calcium chloride (CaCl_2_). The moisture content of soil samples was determined by oven drying 4.0 ± 0.2 g of soil in a tin weigh boat at 60 °C for 48 h (see Method S2 of the Supporting Information). Concentrations of heavy metals (cadmium [Cd], copper [Cu], iron [Fe], lead [Pb], nickel [Ni], and zinc [Zn]) were determined by acid digestion of ∼0.2 g of dried soil in 70% nitric acid (HNO_3_), and then extracts were analyzed using inductively coupled plasma optical emission spectrometry (ICP–OES) analysis using a multi-element standard (see Table S5 of the Supporting Information).

### 2.3 Nitrogen Mineralization Assays

To prepare soil samples for N mineralization assays, two 4.0 ± 0.2 g subsamples were taken from each sample: one for the initial extraction sample (NI) and one for the final extraction sample (NF) and placed in 15 mL tubes. NF samples were incubated in the dark at room temperature for 17 days to allow gas exchange to take place. NI and NF samples were analyzed for nitrate + nitrite concentrations, nitrite only concentrations, and ammonium concentrations using a segmented flow analyzer (Seal Analytical, Ltd., Wrexham, U.K.) to allow for a net rate of nitrification, ammonification, and total N mineralization per day to be calculated from the initial and final concentrations. For samples that produced values that were higher than the upper limit of quantification, 1:5 dilutions with 0.01 M CaCl_2_ solution were run. To analyze the concentrations of nitrate + nitrite, AutoAnalyzer Method G-109-94, ISO 13395, was used from Seal Analytical. To analyze concentrations of ammonium, AutoAnalyzer Method G-102-93, ISO 11732, was used. Concentrations were converted from a mass nutrient per unit volume to a mass nutrient per mass dry soil weight, and then net nitrification, net ammonification, and net total N mineralization rates were calculated (see Method S3 of the Supporting Information).

### 2.4 Quantification of Soil NO_*y*_ Gas Fluxes

A method for NO_*y*_ gas flux analysis from intact cores was developed. One intact core per land-use type was taken for each location and repeated for three seasons, giving a total of 36 samples. This instrument generates one data point per minute for the duration of the 48 h analysis period, giving 2880 data points per sample. Figure S4 of the Supporting Information shows the flux analysis setup. Fluxes of total NO_*y*_, NO, and NO_2_ + NO_*z*_ were analyzed using a Teledyne T200U-NO_*y*_ analyzer (Teledyne, Thousand Oaks, CA, U.S.A.). This system uses a chemiluminescence technique, where NO and ozone (O_3_) react, and the resulting luminescence can be measured to give a direct concentration measurement of NO. NO_*y*_ species other than NO were analyzed separately using the T200U-NO_*y*_ external converter: NO_*y*_ gases were converted to NO by a flow-through molybdenum converter, and resulting NO was then analyzed by the chemiluminescence detector and presented as NO_*y*_–NO measurements, which can also be defined as NO_2_ + NO_*z*_, as in the T200U-NO_*y*_ instrument manual. Conversion efficiency of NO_2_ to NO was greater than 96% over the course of the analyses. Zero air enters the system, from a CG15L purge gas generator [43-1010 (230 V)] and precision air compressor [65-055 (230 V)] (Peak Scientific Instruments). There is a possibility of a small amount of reactive N being present in the zero air as a result of measuring in parts per billion (ppb). Differences between NO and NO_*y*_ concentrations were used to calculate the NO_2_ + NO_*z*_ concentration. The baseline was generated by running an empty flux chamber as a “blank” for 2 h. For flux calculations (defined in Method S4 of the Supporting Information), the flow rate (m^3^/h) out of the flux chamber before and after the measurement period was recorded and an average was taken. The flow rate did not vary by more than 5% over the course of analysis.

Intact cores were first brought to room temperature over 24 h and then normalized to 18–25% gravimetric water content (GWC) by the addition of reagent-grade water. Cores were analyzed for 48 h. To examine the effects of N deposition on the different soil types, a subsequent experiment was performed where 0.39 mg of ammonium nitrate (NH_4_NO_3_) was dissolved in reagent-grade water and added to soil within chambers and gas flux was analyzed for a further 24 h. The amount used was equivalent to 4 months of total average N deposition over the entire U.K. or one season, as reported by Tomlinson et al.^26^ (Figure S6 of the Supporting Information). It should be noted that, as the temperature and water content of these soils have been normalized in these lab-based experiments, future field experiments are required to validate the relevance of these measurements to real-world scenarios.

### 2.5 Analysis of Soil Microbiomes

DNA was extracted from 0.25 g subsamples stored at −80 °C according to the Qiagen DNeasy PowerSoil Kit protocol. DNA concentrations were quantified by the Qubit assay using the protocol of the manufacturer. For 16S community sequencing, targeting bacterial and archaeal species, 16S rRNA gene fragments were amplified from the extracted DNA using primers 515f (5′-GTGCCAGCMGCCGCGGTAA-3′) and 806r (5′-GGACTACHVGGGTWTCTAAT-3′). Amplicons were subjected to 250 bp, paired-end sequencing on Illumina MiSeq Nano, as in the study by Hilton et al.^27^ Abundances of aerobic N cycling genes were assessed by quantitative polymerase chain reaction (qPCR) of DNA extracts on an Agilent Technologies Stratagene Mx2005p qPCR system. Quantitative characterization of the aerobic N cycling community of these samples was appropriate because these surface soils are likely primarily oxic. The specific genes associated with nitrification that we investigated are rate-limiting and, therefore, the most important to consider for N fluxes.^28^ Primers are listed in Table S6 of the Supporting Information. *C*_t_ values were converted to gene copies g^–1^ of soil (see Method S6 of the Supporting Information).

Analysis of fastq files from 16S amplicon sequencing was done using Qiime2 (version 2022.2.0).^29^ Primers were trimmed from sequences, and sequences were demultiplexed using the cutadapt tool. Sequences were denoised, and paired ends were merged using DADA2. Taxonomy was assigned to operational taxonomic units (OTUs) using the Greengenes^30^ full-length 16S rRNA gene database, and mitochondria and chloroplasts were removed. R package phyloseq_1.38.0 was used to create a phyloseq object for the following analysis of phylogenetic data. Functional groups were assigned to OTUs using Functional Annotation of Prokaryotic Taxa (FAPROTAX, version 1.2.6).^31^ Version 1.3.5 of the collapse_table.py script was used with python3 (version 3.8.9 64-bit).^32^

### 2.6 Statistical Analyses

Potential mean NO_*y*_ flux measurements had 36 samples, with 2880 data points per sample. 16S rRNA amplicon sequencing was carried out on 108 samples. All other measured variables had 132 samples. Statistical tests were performed using R (version 4.1.2)^33^ and Julia (version 1.7).^34^ Shapiro–Wilk tests were carried out to test for normality of the data sets. Data showed non-normal distributions; therefore, non-parametric statistical tests were used. Significance of differences between measured variables by sample location, land-use type, and season were tested using Kruskal–Wallis rank sum tests. The effect of each measured variable was first analyzed independently of the others, with potential interactions, and then investigated in cases of significance. *p* values were corrected for multiple comparisons with Dunn’s test using the false discovery rate with the Benjamini–Hochberg method using the R package FSA_0.9.4. Spearman’s rank correlation coefficients were used to test for significant correlations between measured variables. Significant differences were inferred with *p* < 0.05. Potential NO_*y*_ flux measurements generated a value per minute for the duration of the 48 or 24 h continuous measurement period. Hourly means were calculated using Julia, with CSV (version 0.10.3), DataFrames (version 1.3.2), and StatsBase (version 0.33.16). Non-metric multidimensional scaling analysis (NMDS) and analysis of similarity (ANOSIM) were carried out using R packages vegan_2.6-4 and plyr_1.8.8.

To assess the relative importance of soil physicochemical properties, N-cycle-associated microbes, net rates of N cycle processes, land-use type, and human influence on potential mean NO_*y*_ fluxes, we first carried out a factorial analysis of mixed data (FAMD) to condense heavy metal concentrations, road proximity, and sample location into single values that represent contribution to the “human influence” effect. We used R packages FactoMineR_2.7 and factoextra_1.0.7. We then created a structural equation model where soil physicochemical properties, N-cycle-associated microbes, net rates of N cycle processes, land-use type, and human influence were composite fixed effects and seasons were random effects. This analysis was done using the lme4_1.1-31 and piecewiseSEM_2.1.2 R packages. Plots were created using R packages ggplot2_3.3.6, patchwork_1.1.2 and vcd_1.4-11.

## 3 Results

### 3.1 Potential N Mineralization Rates

**Figure 2:**
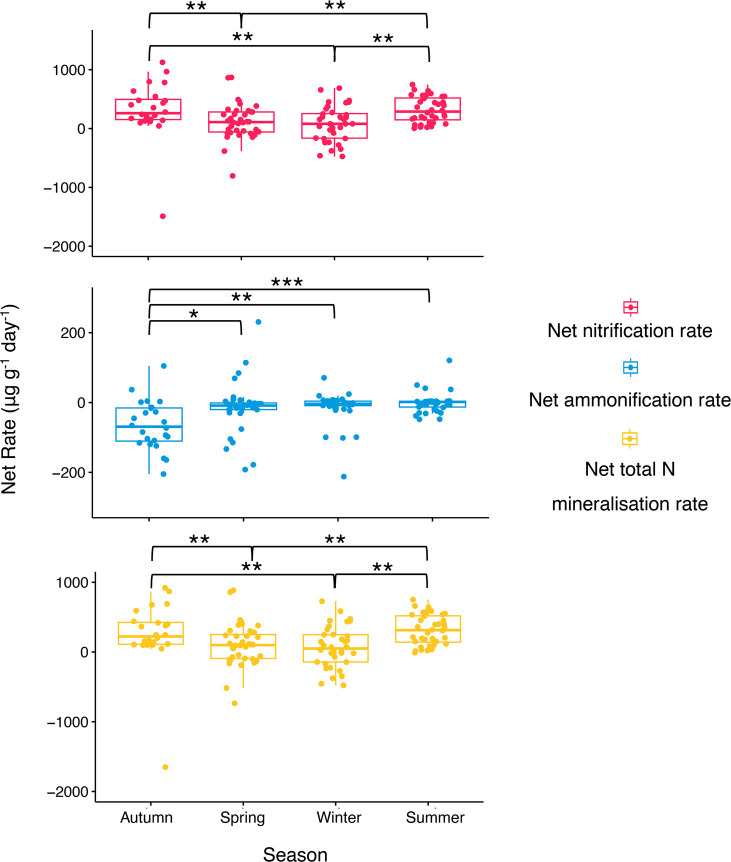
Effect of season on net rates of nitrification, ammonification, and N mineralization from soils. Concentrations of NO_3_^–^, NO_2_^–^, and NH_4_^+^ were analyzed before and after a 17 day incubation period using a segmented flow analyzer and used to calculate net rates of nitrification, ammonification, and N mineralization per gram of soil per day. Significance lines indicate results from Kruskal–Wallis with Dunn’s tests, where net rates of nitrification, ammonification, and N mineralization were compared between seasons (∗, <0.05; ∗∗, <0.01; and ∗∗∗, < 0.001). *N* = 132.

Concentrations of available NO_3_^–^ as well as net rates of nitrification, ammonification, and total N mineralization were seasonally dependent, with overall less influence from land use or anthropogenic sources (Figure 2). Kruskal–Wallis tests revealed significant differences in seasons between total net N mineralization rates (*p* < 0.001), net nitrification rates (*p* < 0.01), and net ammonification rates (*p* < 0.001). Net N mineralization rates were highest from summer samples; net nitrification rates were highest from autumn samples; and net ammonification rates were highest in spring. Net nitrification rates were significantly affected by land-use type (*p* < 0.05), with the highest rates from woodland soils and the lowest rates from agricultural soils. We observed no significant differences in net nitrification, net ammonification, nor total net N mineralization rates between sample locations. Available NO_3_^–^ and NH_4_^+^ concentrations showed seasonal differences (*p* < 0.001, for both). The highest concentration of NH_4_^+^ was measured from autumn samples, and the lowest concentration was measured from winter samples (see Figure S3 of the Supporting Information). Despite differences in agricultural management practices, there was no significant difference in available NO_3_^–^ or NH_4_^+^ in agricultural soils between locations. Using Spearman’s rank correlations, we observed a significant positive correlation between the soil moisture content and total net N mineralization rate as well as net ammonification rate (*p* < 0.01). There was a statistically significant positive correlation between pH and total net N mineralization rate (*p* < 0.01).

### 3.2 NO_*y*_ Fluxes

**Figure 3:**
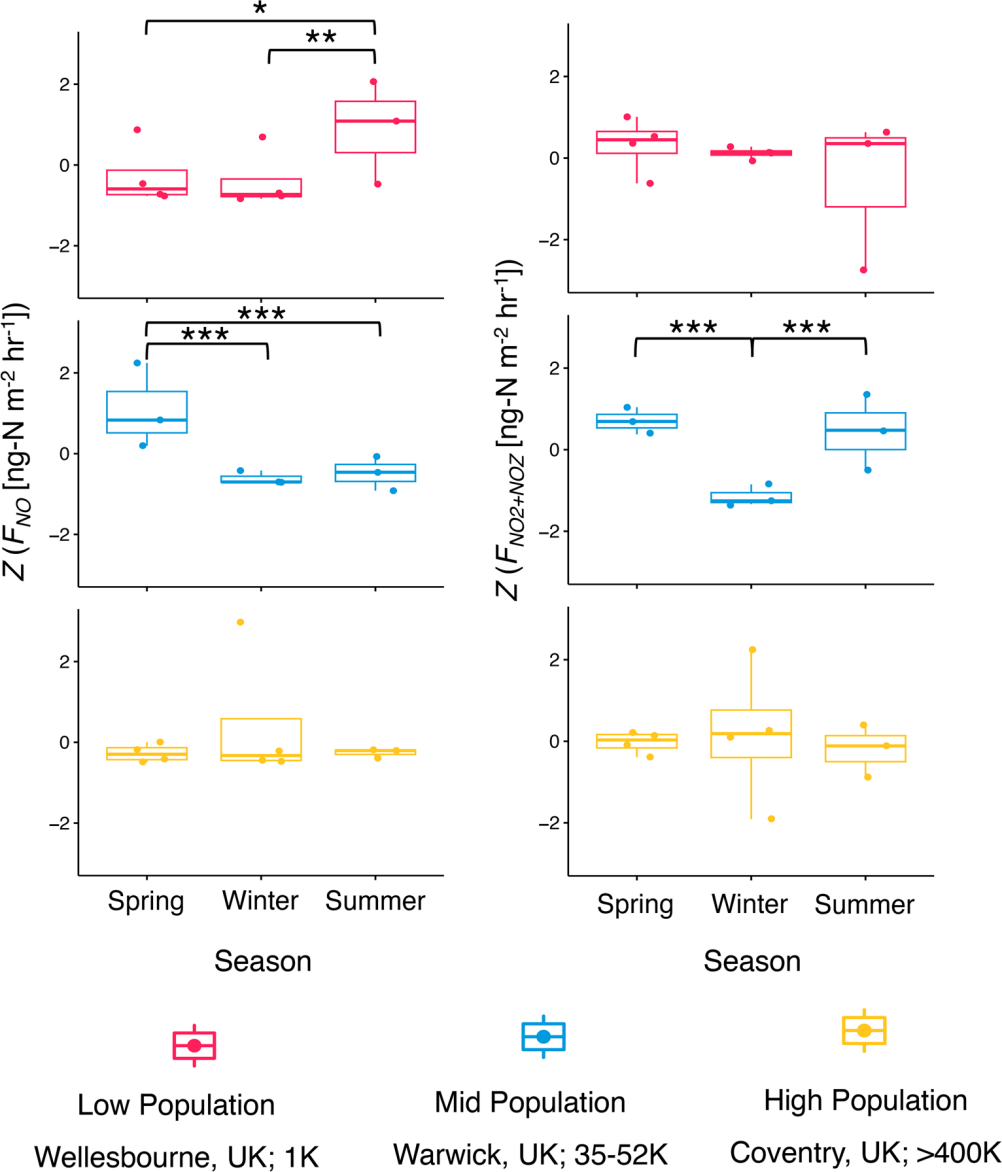
Effect of the season (spring, winter, and summer) and location (“low population, Wellesbourne, U.K., <1000”, “mid-population, Warwick, U.K., 35 000–52 000”, and “high population, Coventry, U.K., >400 000”) on potential mean fluxes of NO (*F*_NO_) and NO_2_ + NO_*z*_ (*F*_NO_2_ + NO_*z*__). *Y* axes are *Z*-transformed. Fluxes were measured with a chemiluminescence technique using a Teledyne T200U instrument. *N* = 36. Significance lines indicate significant differences in fluxes between seasons (∗, <0.05; ∗∗, <0.01; and ∗∗∗, <0.001).

**Figure 4:**
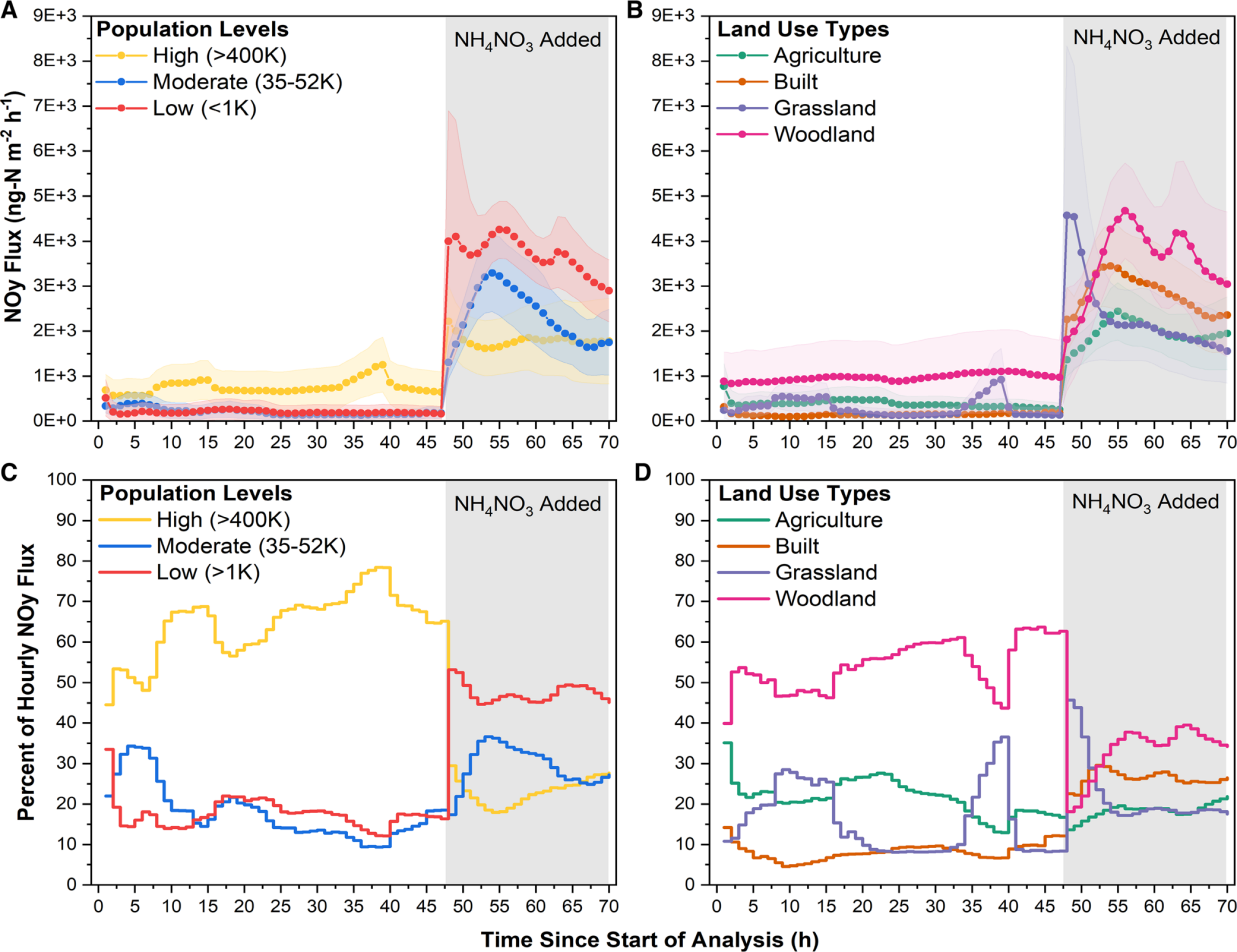
(A) Effect of the addition of NH_4_NO_3_ to simulate 3 months (one season) worth of N deposition to U.K. soils on potential mean fluxes of total NO_*y*_ (*F*_NO_*y*__) between locations (“low population, Wellesbourne, U.K., <1000”, “mid-population, Warwick, U.K., 35 000–52 000”, and “high population, Coventry, U.K., >400 000”). (B) Effect of the addition of NH_4_NO_3_ to simulate 3 months (one season) worth of N deposition to U.K. soils on potential mean fluxes of *F*_NO_*y*__ between land-use types (agricultural, woodland, grassland, and built). Fluxes were measured with a chemiluminescence technique using a Teledyne T200U instrument for 48 h previous to N addition and 24 h after N addition. (C) Relative contribution (%) of each location to summed *F*_NO_*y*__ for each hour, before and after N addition. (D) Relative contribution (%) of each land use to summed *F*_NO_*y*__ for each hour, before and after N addition.

Mean flux values and standard errors can be found in Table S4 of the Supporting Information. Kruskal–Wallis and Dunn’s tests show no significant differences in potential mean NO_*y*_ fluxes over 48 h (*F*_NO_*y*__) or potential mean NO fluxes over 48 h (*F*_NO_) between land-use types. These results also exhibit no significant differences in *F*_NO_*y*__ or *F*_NO_ between locations with different human populations. However, potential mean NO_2_ + NO_*z*_ fluxes (*F*_NO_2_ + NO_*z*__) were affected significantly by land-use type (*p* < 0.05), with the highest fluxes from grass-dominated soils (Figure S5 of the Supporting Information). *F*_NO_ and *F*_NO_2_ + NO_*z*__ showed significant temporal variabilities (*p* < 0.01 and *p* < 0.05, respectively; Figure 3). *F*_NO_ values were highest from winter samples. *F*_NO_2_ + NO_*z*__ values were significantly more negative in summer and winter samples than spring samples, indicating possible uptake of NO_*y*_ into the soil from the zero-air source (see section 2.4). No measured mean fluxes had an association with the soil pH or soil moisture content. Baseline *F*_NO_*y*__ (before N addition) were positively correlated with the human population of sampling sites (Figure 4A). After N addition, this correlation was reversed, and the highest *F*_NO_*y*__ values were measured in samples from low human population sites. Baseline *F*_NO_*y*__ values were highest from woodland sites compared to other land-use types (Figure 4B). After N addition, woodland soils still demonstrated the highest *F*_NO_*y*__. Panels C and D of Figure 4 show the percentage contribution of each sample location and land-use type, respectively, to the total *F*_NO_*y*__ for each hour of the measurement period. Samples from locations with high human populations dominate the baseline *F*_NO_*y*__ but contribute the least to the total flux after N addition.

### 3.3 16S Microbial Communities

**Figure 5:**
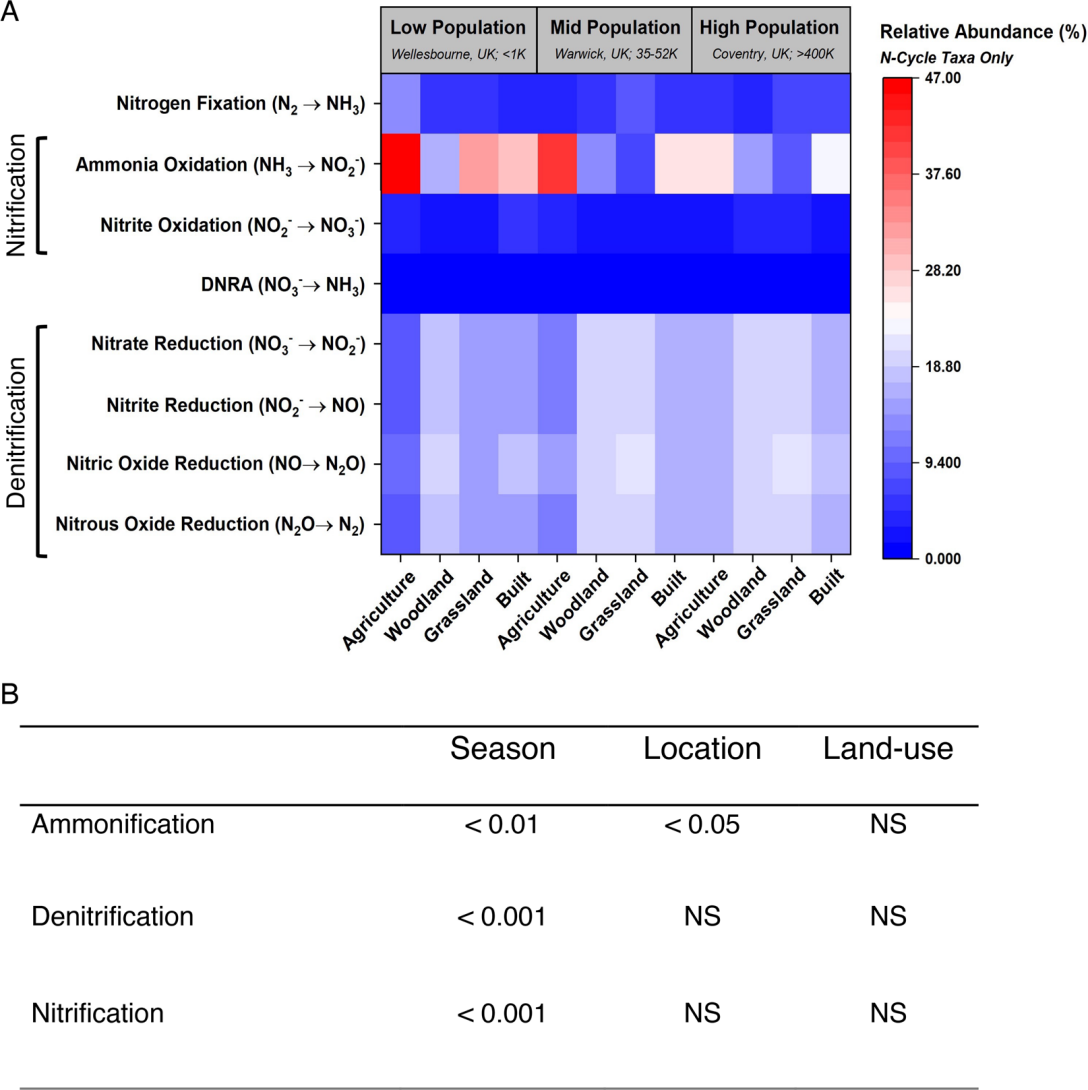
(A) Heat map of relative abundances of microbial orders associated with nitrogen (N) cycling processes compared between locations (“low population, Wellesbourne, U.K., <1000”, “mid-population, Warwick, U.K., 35 000–52 000”, and “high population, Coventry, U.K., >400 000”) and land use (agricultural, woodland, grassland, and built). Microbial communities were quantified with 16S rRNA amplicon sequencing on Illumina MiSeq Nano. *N* = 108. (B) Table of *p* values obtained from Kruskal–Wallis rank sum tests, where relative abundances of N cycling microbial taxa were compared between locations (“low population, Wellesbourne, U.K., <1000”, “mid-population, Warwick, U.K., 35 000–52 000”, and “high population, Coventry, U.K., >400 000”), land-use types (agricultural, woodland, grassland, and built), and seasons (spring, winter, and summer). NS indicates non-significant results.

16S rRNA amplicon sequencing results showed that Fisher’s α diversity (Figure S8 of the Supporting Information) of the bacterial and archaeal communities was not significantly different between samples from different land-use types or locations. Non-metric multidimensional scaling (NMDS; Figure S9 of the Supporting Information) and analysis of similarity (ANOSIM) showed that composition differed with human population sizes (*p* < 0.001). Significant differences were also apparent between samples from different land-use types. In particular, agricultural and built samples were each significantly different to all other land-use types (*p* < 0.001). Analysis of only the N-cycling-associated microbial community revealed significant differences in relative abundances between sample locations and land-use types for (1) nitrification-associated orders, Nitrososphaerales, Nitrosomonadales, and Cenarchaeales (*p* < 0.001, for all three), and for (2) denitrification-associated orders, Rhizobiales, Rhodobacterales, Clostridiales, Rubrobacterales, Gaiellales, and Bacillales (*p* < 0.05, *p* < 0.05, *p* < 0.001, *p* < 0.05, *p* < 0.05, and *p* < 0.001, respectively; Figure 5A). N-cycle-associated microbial orders Rhizobiales, Rhodobacterales, and Opitutales had significantly different abundances relative to the total N cycling community between seasons (*p* < 0.05, *p* < 0.05, and *p* < 0.001, respectively). The abundance of Gaiellales relative to the total N community was significantly negatively associated with *F*_NO_2_ + NO_*z*__ (*p* < 0.01). Cenarchaeales are an order of archaea involved with ammonia oxidation, the first stage of nitrification. We found that the abundance of Cenarchaeales relative to the total N community was significantly positively associated with *F*_NO_*y*__ and *F*_NO_ (*p* < 0.05, for both). Abundances of Rhizobiales, Rhodobacterales, and Opitutales relative to the total N community were significantly different between seasons (*p* < 0.05, *p* < 0.05, and *p* < 0.001, respectively), with the highest abundances in winter for all three orders. Abundances of Nitrospirales, Rhizobiales, Rhodobacterales, and Cenarchaeales relative to the total N community were significantly negatively correlated with the soil moisture content (*p* < 0.01, *p* < 0.001, *p* < 0.001, and *p* < 0.05, respectively), whereas the abundance of Nitrosomonadales was significantly positively correlated with the soil moisture content (*p* < 0.01). Increased relative abundances of Cenarchaeales and Rhizobiales were correlated with lower pH (*p* < 0.01 and *p* < 0.001, respectively). Increased relative abundances of Rubrobacterales, Clostridiales, Rhodobacterales, and Nitrososphaerales were significantly associated with higher pH (*p* < 0.001, *p* < 0.05, *p* < 0.05, and *p* < 0.001, respectively). Following functional annotation of OTUs using the FAPROTAX database, we found no significant differences in the abundance of taxa with potential to carry out nitrification and denitrification, relative to each other, between locations or land-use type. There was a significant difference in the abundance of taxa with potential to carry out ammonification between low- and high-population sample locations (*p* < 0.05; Figure 5B).

**Figure 6:**
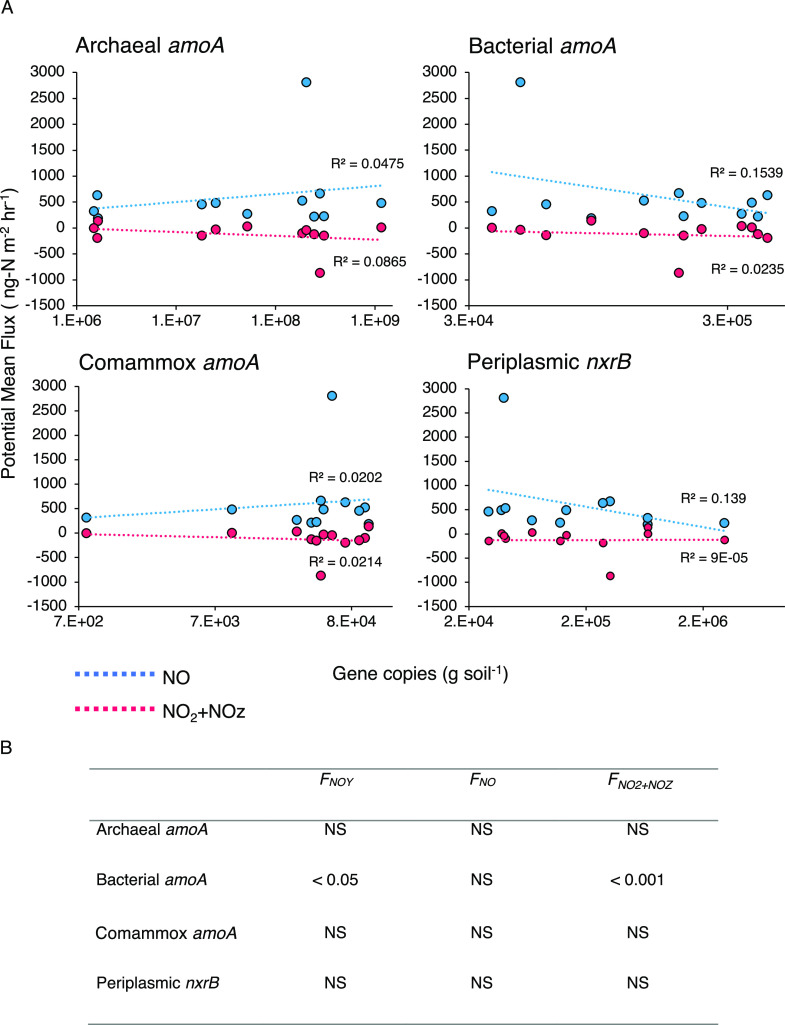
(A) Copies of bacterial, archaeal, and comammox *amoA* and periplasmic *nxrB* genes per g of soil obtained from quantitative PCR plotted against potential mean fluxes of NO (*F*_NO_) and NO_2_ + NO_*z*_ (*F*_NO_2_ + NO_*z*__). Fluxes were measured with a chemiluminescence technique using a Teledyne T200U instrument. (B) Table of *p* values obtained from Spearman’s rank correlation coefficients testing correlations between gene copy numbers and potential mean fluxes of NO_*y*_ (*F*_NO_*y*__), *F*_NO_, and *F*_NO_2_ + NO_*z*__. NS indicates non-significant results. *N* = 48.

Copies g^-1^ of soil for bacterial *amoA* and *nxrB* genes were significantly different between seasons (*p* < 0.001, for both), with the highest copies g^–1^ of soil in autumn and spring samples, respectively (Figure 6A). No quantified genes were significantly different between land-use types or sample locations. Copies g^–1^ of soil of bacterial *amoA* were significantly positively correlated with *F*_NO_*y*__ and *F*_NO_2_ + NO_*z*__ (*p* < 0.05 and *p* < 0.001, respectively; Figure 6B). Although one particularly high *F*_NO_ value may be driving this significance, further investigation determined that correlations remain significant even when this value is omitted. Copies g^–1^ of soil *nxrB* were significantly positively correlated with the soil moisture content (*p* < 0.05).

### 3.4 Heavy Metal Concentrations

Concentrations of heavy metals showed significant variability between land-use type, sample location, and season (Figure S10 of the Supporting Information). Concentrations of Cd and Pb were significantly positively correlated with the human population gradient of the samples, with the highest concentrations recorded in high human population samples (0.025 and 3.19 mg g^–1^ of soil, respectively) and the lowest concentrations recorded in low human population samples (−0.027 and 0.070 mg g^–1^ of soil, respectively) and significant differences between these locations. Low human population samples contained the highest concentration of Fe (1077.25 mg g^–1^ of soil), of which the concentration was inversely correlated with the human population gradient. Pb was the only element to be significantly affected by both location and land-use type (*p* < 0.001). Concentrations of Zn were significantly different between autumn and spring samples and between summer and spring samples (*p* < 0.01), with the highest concentrations in spring samples. Cu, Ni, Pb, and Zn were all significantly affected by land-use type. Cu, Ni, and Zn were significantly higher in built soils (*p* < 0.05). Pb was highest in woodland soils (*p* < 0.01). Concentrations of Cu, Cd, and Pb were significantly positively correlated with proximity of sampling sites to the closest road (*p* < 0.001, for all; Figure S11 of the Supporting Information). Concentrations of Cd and Pb had a significant negative correlation with pH values (*p* < 0.001, for both). Concentrations of Fe had a significant positive correlation with pH values (*p* < 0.001). Concentrations of Cu, Pb, Ni, and Zn were significantly negatively correlated with the soil moisture content (*p* < 0.001, *p* < 0.001, *p* < 0.01, and *p* < 0.01, respectively). Concentrations of Pb and Ni were significantly correlated with the potential mean *F*_NO_2_ + NO_*z*__.

### 3.5 Structural Equation Modeling

**Figure 7:**
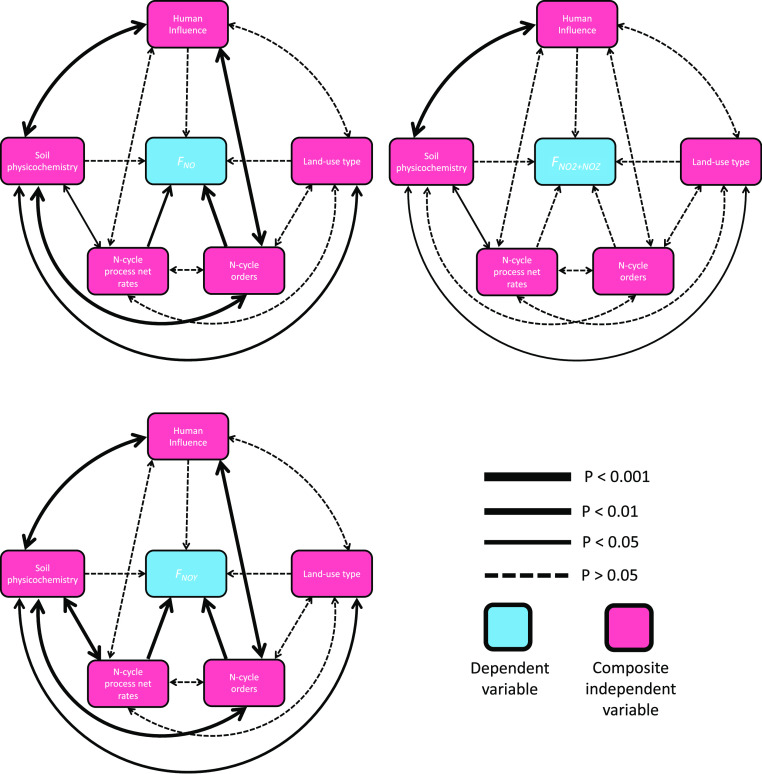
Structural equation modeling to ascertain effects of the measured variable on fluxes of NO (*F*_NO_), NO_2_ + NO_*z*_ (*F*_NO_2_ + NO_*z*__), and NO_*y*_ (*F*_NO_*y*__). Fixed effects: composite soil physicochemical properties (pH and moisture content), *N* = 132; composite relative abundances of nitrogen (N)-cycle-associated microbial taxa, *N* = 108; composite net rates of N cycle processes (nitrification, ammonification, and N mineralization), *N* = 132; land-use type (agricultural, woodland, grassland, and built), *N* = 132; and human influence, *N* = 132. The season (autumn, spring, winter, and summer) is a random effect. The “human influence” variable is the result of a factorial analysis of mixed data (FAMD) to condense heavy metal concentrations from ICP–OES analysis, road proximity, and sample location (“low population, Wellesbourne, U.K., <1000”, “mid-population, Warwick, U.K., 35 000–52 000”, and “high population, Coventry, U.K., > 400 000”) into single values.

Structural equation modeling showed that N cycle process net rates and N cycle orders had the most significant effect on *F*_NO_*y*__ (*p* < 0.001, for both; Figure 7). The human influence composite variable had significant associations with soil physicochemistry and N cycle orders (*p* < 0.001, for both). Soil physicochemistry was significantly associated with most other composite variables, with N cycle process net rates (*p* < 0.001), with N cycle orders (*p* < 0.001), and with land-use type (*p* < 0.01). There were similar effects seen for *F*_NO_. *F*_NO_ was significantly affected by N cycle process net rates (*p* < 0.01) and N cycle orders (*p* < 0.001). However, the interaction between soil physicochemistry and N cycle process net rates was slightly less significant (*p* < 0.05) in this model compared to the *F*_NO_*y*__ model. No composite variable demonstrated statistically significant effects on *F*_NO_2_ + NO_*z*__. Soil physicochemistry was significantly associated with land-use type (*p* < 0.05), N cycle process net rates (*p* < 0.05), and human influence (*p* < 0.001). Human influence and N-cycle process net rates had significant interactions with at least one other variable in all models. *R* values for these mixed effects models can be found in Figure S13 of the Supporting Information.

## 4 Discussion

### 4.1 Anthropogenically Influenced Soils Are Sources of Enhanced NO_*y*_ Emissions

N deposition (kg of N ha^–1^ year^–1^) in the locations with high human populations is greater than in the locations with mid- and low human populations. Soil samples from locations with high human populations typically have a higher *F*_NO_*y*__, indicating an anthropogenic impact on these soils. However, after the addition of N that equated to approximately one season of deposition to the intact soil cores, samples from locations with high human populations appear to be buffered against increasing *F*_NO_*y*__. We suggest that *F*_NO_*y*__ from soils from locations with a high human population may be due to abiotic processes more so than locations with lower human populations. The addition of N stimulates microbial activity and biotic mechanisms of *F*_NO_*y*__ production; however, more anthropogenically influenced soils seem to have less capacity for microbially driven N cycling. This could be due to suppressed microbial activity from polluted soils, related to the higher metal concentrations measured from soils from locations with high human populations. We also identified lower relative abundance of microbes associated with ammonia oxidation in soils from locations with high human populations. AOA and AOB have been found to be affected by increased heavy metal pollution, including Cd, Cu, and Pb, which were found in this study to be significantly positively correlated with the human population.^45^

One of the sample sites, a woodland in the high human population area of Coventry, had unusually high concentrations of lead. This sample had an acidic pH of 3.87 as an average over the seasons, which is lower than what would be expected from soils of this type and potentially as a result of the accumulation of metals at the soil surface. Interestingly, there was a significant increase in *F*_NO_ from this sample. This sample site is 23 m from a busy road, with idle traffic often present. Studies that investigate “urban” soils may not consider the gradients of urbanization that can be found, instead pooling data to compare to “non-urban” soils. Some sample sites are therefore sufficiently removed from proximity to roads and anthropogenic activity that they are buffered from drastic changes to physiochemical properties and subsequent changes to N cycle processes.^35,36^ We suggest that this leads to studies missing so-called NO_*y*_ “hotspots” from those soils most severely affected by human influence, such as this Coventry woodland sample. This work demonstrates the need to examine the most heavily anthropogenically influenced soils to capture potential NO_*y*_ hotspots that may affect future climate modeling.

### 4.2 Microbial NO_*y*_ Production Is Dominated by AOB Taxa

The abundances of nitrification-associated taxa were more significantly affected by land-use type than denitrification-associated taxa; however, the differences in functional potential between land-use types does not seem to translate to N cycle gene quantity and potential activity. This could be due to this work being carried out *ex situ*, where the actual activity of the microbial community was not representative of soils in the field. We found that more copies of g^–1^ of soil of bacterial *amoA* were associated with increased *F*_NO_*y*__, driven by *F*_NO_2_ + NO_*z*__. It has been hypothesized that AOA species are the primary drivers of nitrification rates in soils as a result of the high levels of AOA compared to AOB. However, it has been reported that this does not translate into enhanced effects on *F*_NO_*y*__, and AOB species are the primary producers of *F*_NO_*y*__ in forest soils.^6^ While there were no statistically significant correlations between abundances of any ammonia oxidizing taxa and *F*_NO_*y*__ found in this work, increased relative abundances of AOA were not associated with increased *F*_NO_*y*__, whereas abundances of AOB and comammox taxa were. These data suggest that NO_*y*_ production across soils from all sample sites and not only woodland soils is primarily driven by AOB.

### 4.3 Spatiotemporal Trends in Soil-Sourced N Fluxes

The experimental design of investigations into NO_*y*_ fluxes from soils to date usually take soils from a single or few time points, focusing instead on a large sample area.^16,37^ Those that include soils collected over time are likely to pool the data into sample locations.^38^ Here, we show the importance of taking the temporal variability of NO_*y*_ gas fluxes into account during this type of investigation to build a more accurate picture of soil-sourced atmospheric pollution. In this study, we report seasonal variation to be an important and currently underestimated predictor of net rates of nitrification, ammonification, and total N mineralization, which can lead to NO_*y*_ fluxes from soil. Here, the differences in NO_*y*_ fluxes seen between sample sites were based on current land-use and vegetation cover, and we cannot make in depth assumptions based on the effects of historical land use. However, we suggest a predictability of soil physicochemical properties based on the current land-use type and human population, with consequences for N fluxes in these soils.

We suggest that the increased NO emissions from woodland soils in this study are due to organic matter input from leaf fall in autumn, leading to an increased concentration of available NO_3_^–^, with the consequential flux increase seen in winter. This could be due to the increased abundance of N cycle microbes associated with denitrification (Rhizobiales, Rhodobacterales, and Opitutales) in winter samples, through which NO can be produced. It would be expected that the characteristic low temperatures of winter would lead to reduced microbial activity.^39,40^ However, here, we are measuring potential N fluxes rather than in the field; therefore, results may not be representative of *in situ* microbial activity. With increased abundance of N cycle microbes, we would expect to also see increased rates of net N cycle processes, which we do not, suggesting that an abiotic component may also contribute to increased NO emissions. We suggest chemodenitrification, the chemical decomposition of NO_2_ particularly at low pH, to be a probable mechanism for NO formation in this case. As a result of decomposition of organic matter by heterotrophs, it is common for woodland soils to be relatively acidic compared to other land-use types, and excess H^+^ protons may have consequences for N cycle process pathways that lead to NO_*z*_ emissions. The significantly more negative *F*_NO_2_ + NO_*z*__ that we report from woodland samples suggests an increased uptake of NO_2_ that then reacts with H^+^ as a likely mechanism of HONO, a NO_*z*_ species, formation.^41^ These data would also suggest that the NO_*z*_ species produced are not emitted as readily from these woodland soils.

Fertilization of agricultural fields is a seasonal N input into soils. Few plants require winter fertilization, even if they are winter-flowering.^42^ We suggest that enhanced *F*_NO_ and *F*_NO_2_ + NO_*z*__ from agricultural soils in spring samples compared to other seasons are due to input of available N from fertilizers during this growing season, which causes a higher rate of nitrification. Measured concentrations of available NH_4_^+^ from agricultural soils were also the highest in spring samples. The high abundance of microbial orders with functional potential to carry out nitrification and the high number of copies of the *amoA* gene in agricultural soils compared to other land-use types reinforce this theory. Initially measured concentrations of NO_3_^–^ are highest in summer samples, suggesting that application of fertilizers in spring may lead to a legacy of higher N cycle microbial potential in the following seasons. These data clearly show that investigations into NO_*y*_ fluxes from soils must consider this seasonal variation, particularly because human influence on soils can fluctuate throughout a year. Here, we demonstrate seasonally altered microbial community composition influencing biotic reactive N fluxes. We also see potential for seasonal effects on abiotic mechanisms of NO_*y*_ formation, although this would require further investigation to elucidate.

Yan et al. report lowest pH values in woodland soils compared to those from “farmland”, “grassland”, or “bareland” and the highest in “farmland”.^43^ This is reflected in this data set; pH of samples from agricultural samples were significantly higher than all other land-use types, particularly grass-dominated samples. Agricultural samples also had significantly higher moisture contents than the other land-use types, potentially as a result of irrigation practices. In this data set, we found that grass-dominated soils tended to be a “middle ground” in terms of the measured variables, suggesting grassland to be a buffered soil type, capable of withstanding pressures from chemical and physical disturbances. The prevailing mindset is that regeneration of woodlands is one of the most effective ways to combat increasing greenhouse gas (GHG) emissions;^44^ however, more recently, it has been suggested that many reforestation projects do not take into account the GHGs produced by the creation of woodlands and from the woodlands once they are established.^45^ This study and those such as Dass et al. agree that, if conversion of land to woodland comes at the expense of losing grassland habitats, this is also a loss of important GHG sinks.^46^

### 4.4 Structural Equation Modeling (SEM) Reveals Direct and Indirect Influences on Reactive N Fluxes on a Landscape Scale

Mixed effect models suggest that the soil microbial community (and therefore biotic N cycling capacity) is the most important direct effector of NO_*y*_ emissions, particularly in the case of NO. We suggest that abiotic N cycle processes are indirect effectors of reactive N fluxes in soils. The lack of association between N cycle orders and N cycle process net rates further suggests an abiotic component of soil reactive N fluxes. Human influence was a strong effector of soil physicochemical properties in all models, which is consistent with other interactions, such as the significant correlation between the human population of the sample locations and pH values. This effect could also be due in part to the outlying Coventry woodland sample as mentioned in section 4.1. Significant interactions between soil physicochemistry and land-use type are consistent with results discussed in section 4.3, and we suggest these interactions to be indirect effectors of reactive N fluxes on a landscape scale. The composite variable human influence demonstrated more significant effects with other variables than land-use type, suggesting that changes to the soil system as a result of anthropogenic activities contribute more to reactive N fluxes from soils than land-use type.

This work is a unique investigation of reactive N fluxes on a landscape scale, taking a comprehensive set of land-use types, human influence, and seasonality into account to determine large-scale heterogeneity. This work provides a baseline for hypothesis generation for study in this area and highlights the critical importance of the spatial and temporal variety of soil samples in future studies of reactive N fluxes. Following this work, smaller scale heterogeneity of reactive N fluxes and other soil characteristics should be investigated to build an accurate data set for use in environmental modeling and climate change predictions.

## Data Availability

Data are available at Purchase, Megan (2023), “Spatiotemporal variations of soil reactive nitrogen oxide fluxes across the anthropogenic landscape”, Mendeley Data, v1; doi: 10.17632/xykvwd3twg.1. The code is available at https://github.com/MeganPurchase/Spatiotemporal_NOy_Purchase23.git.

## Supporting Information Available

Additional experimental details, methods, and results, including diagrams of experimental setups (PDF)
