# Supplementary material for: Spatiotemporal Variations of Soil Reactive Nitrogen Oxide Fluxes across the Anthropogenic Landscape
Source: Environ Sci Technol. 2023 Oct 19;57(43):16348–60. doi: 10.1021/acs.est.3c05849 (PMC10620987; doi:10.1021/acs.est.3c05849)
Supplement: Supplementary file 1 — es3c05849_si_001.pdf [file es3c05849_si_001.pdf]

Supplemental Information for

Spatiotemporal variations of soil reactive nitrogen oxide fluxes across the anthropogenic landscape

*Megan L Purchase[1], Gary D Bending[1], Ryan M Mushinski[1]

[1]School of Life Sciences, University of Warwick, Coventry, CV4 7AL, UK

*Corresponding author; m.purchase@warwick.ac.uk

Summary
21 pages, 6 methods, 6 tables, 13 figures.

Table of Contents

Methods……………………………………………………………..………..….2

S1. Agricultural Management Practices of Sample Sites…………………………..2

S2. Soil Physiochemical Properties……..………………………………………....2

S3. N mineralisation Assay Calculations……………………………………………2

S4. NOy Flux Analysis Details………..…...………..……………………………....3

S5. Details of Standards for ICP-OES……………………………………………...3

S6. Gene Copy Number Calculations……………………………………………...4

Tables…………………………………………………………………………………....4

Table S1. Site Details………………………………………………………...…4

Table S2. Meteorological Details……………………………………...………6

Table S3. Physicochemical Analyses Significance Values………………………6

Table S4. Mean Values and Standard Errors of Flux Measurements…….……7

Table S5. ICP-OES Standards……………………………………………………8

Table S6. Quantitative PCR Primers…………………………………….……8

Figures………………………………………………………………………………..…9

Figure S1. Map of Sampling Sites…………………………………………….……9

Figure S2. Soil Physicochemical Properties……………………………………...10

Figure S3. Available NO_3^- and NH_4^+ in Agricultural Soils……….…………..11

Figure S4. NOy Flux Analysis Setup…………………………..………….12

Figure S5. Potential Mean NOy Fluxes……………………………………………13

Figure S6. Observed Dry and Wet Total Nitrogen Deposition……..…………14
Figure S7. Relative Abundance of Total Microbial Community……………....15
Figure S8. Fisher's Alpha Diversity Index….…………………………...……….16
Figure S9. Non-metric Multidimensional Scaling Analysis…………………….17
Figure S10. Heavy Metal Concentrations……………..…………………...…..18
Figure S11. Effect of Road Proximity on Heavy Metal Concentrations……….19
Figure S12. Simulated N Deposition Continuous Flux Measurements……….20
Figure S13. Structural Equation Modelling R Values…………………….….21

Methods

S1. Agricultural Management Practice of Sample Sites

Agricultural sites in Coventry are categorised as "mixed farming", with a mix of livestock and arable crops. Agricultural sites in Wellesbourne are used for arable crops, including winter wheat and double sown barley. Coventry and Wellesbourne agricultural sites are managed conventionally with application of manufactured nitrogen fertiliser averaging ~98 Kg/ha/year from 2012-2022 Agricultural sites in Warwick are used for growing of cereals (except rice), leguminous crops and oil seeds. Sites are managed using regenerative farming practices and organic nitrogen fertiliser averaging ~9 Kg/ha/year from 2012-2022. (Data source: UK Government National statistics: Fertiliser usage on farms in England sourced from the Farm Business Survey from Department for Environment, Food & Rural Affairs).

S2. Soil Physicochemical Properties

Soil moisture content was calculated as below:

$$\frac{(dry\ weight + tin\ weight) - (tin\ weight)}{(wet\ weight + tin\ weight) - (tin\ weight)} = dry\ weight{:}\ wet\ weight$$

S3. N mineralisation Assay Calculations

Concentrations were converted from a mass nutrient per unit volume to a mass nutrient per mass dry soil weight using the below calculations:

$$\frac{[NO_3^-]mg}{L} * \frac{extraction\ vol.\,(L)}{soil\ wet\ weight\ (g) * (dry\ weight{:}\ wet\ weight)} * \frac{1000\mu g}{1\ mg} = [NO_3^-]\,\frac{\mu g}{g\ dw}$$

$$\frac{[NH_4^+]mg}{L} * \frac{extraction\ vol.(L)}{soil\ wet\ weight\ (g) * (dry\ weight{:}\ wet\ weight)} * \frac{1000\mu g}{1\ mg} = [NH_4^+]\frac{\mu g}{g\ dw}$$

Net nitrification and net ammonification rates were calculated:

$$\frac{([NO_3^- \frac{\mu g}{g\ dw}]_{final} - [NO_3^- \frac{\mu g}{g\ dw}]_{initial})}{incubation\ time\ (days)} = Nitrification\ rate(\mu g\ NO_3^- \cdot g^{-1} \cdot day^{-1})$$

$$\frac{([NH_4^+ \frac{\mu g}{g\ dw}]_{final} - [NH_4^+ \frac{\mu g}{g\ dw}]_{initial})}{incubation\ time\ (days)} = Ammonification\ rate(\mu g\ NH_4^+ \cdot g^{-1} \cdot day^{-1})$$

N Mineralisation rates were calculated:

$$\frac{(\Sigma\ inorg.N_{final} - \Sigma\ inorg.N_{initial})}{incubation\ time\ (days)} = N\ min.rate\ (\mu g\ inorganic\ N \cdot g^{-1} \cdot day^{-1})$$

S4. NOy Flux Analysis Setup Details

Soil flux chambers were constructed from unplasticised polyvinyl chloride (PVC-U) tubes (diameter = 7cm, height = 20cm), with PVC-U caps. Chambers were made airtight using adjustable steel O-rings. Tubing and tube connectors are made of non-reactive polytetrafluoroethylene (PTFE) (**Fig. S4**). Fluxes (F_{soil}) of NOy, NO, and NO_2+NOz were calculated as below:

$$F_{soil} = (Conc._{soil} - Conc._{blank})x\ flow\ x\ (\frac{Chamber\ volume}{Soil\ surface\ area})$$

S5. Details of Standards for ICP-OES

ICP multi-element standard solution IV from Merck Certipur® line was used for this analysis, containing 23 elements at 1000mg/l in diluted nitric acid (HNO_3): Ag, Al, B, Ba, Bi, Ca, Cd, Co, Cr, Cu, Fe, Ga, In, K, Li, Mg, Mn, Na, Ni, Pb, Sr, Tl, Zn. The standard solution was diluted using 7% HNO_3 to give the concentrations in **Table S4**.

S6. Gene Copy Number Calculations

Copies of standards were calculated as below, then plotted against cycle threshold (ct) values.

$$\frac{Standard\ quantity\ (ng/\mu l)\ *\ 6.022x10^{23}}{product\ size\ (bp)\ *\ 1x10^{9} *\ 660}$$

Line equations ($y = mx + c$) were used to calculate copy numbers of samples per g soil as below:

$$\frac{10^{(m*ct+c)} * extraction\ vol.(\mu l)}{soil\ dry\ weight\ (g)}$$

Tables

Table S1. Site Details. Details of sample sites and soil characteristics.

Human population	Land use	Coordinates	Elevation (ft)	Parent material	Soil texture
**Wellesbourne**	Agriculture	N 52° 12' 33" W 1° 36' 25"	150	Riverine clay, sands and gravels	Clay, sandy loam
**Wellesbourne**	Agriculture	N 52° 12' 22" W 1° 36' 24"	160	River terrace sand/gravel	Sandy loam
**Wellesbourne**	Agriculture	N 52° 12' 19" W 1° 36' 18"	170	River terrace sand/gravel	Sandy loam
**Wellesbourne**	Woodland	N 52° 12' 40" W 1° 36' 37"	130	Riverine clay, sands and gravels	Clay, sandy loam
**Wellesbourne**	Woodland	N 52° 12' 43" W 1° 36' 21"	150	River terrace sand/gravel	Sandy loam
**Wellesbourne**	Woodland	N 52° 12' 13" W 1° 35' 53"	160	River terrace sand/gravel	Sandy loam
**Wellesbourne**	Grassland	N 57° 12' 39" W 1° 36' 28"	170	River terrace sand/gravel	Sandy loam
**Wellesbourne**	Grassland	N 52° 12' 44" W 1° 36' 22"	150	River terrace sand/gravel	Sandy loam
**Wellesbourne**	Grassland	N 52° 12' 30" W 1° 36' 35"	160	River terrace sand/gravel	Sandy loam
**Wellesbourne**	Built	N 52° 12' 39" W 1° 36' 5"	160	River terrace sand/gravel	Sandy loam
**Wellesbourne**	Built	N 52° 12' 39" W 1° 36' 10"	150	River terrace sand/gravel	Sandy loam

**Wellesbourne**	Built	N 52° 12' 40" W 1° 36' 17"	150	River terrace sand/gravel	Sandy loam
**Warwick**	Agriculture	N 52°19'00" W 1°28'40"	260	Siltstone, sandstone	Silty loam, silt, sand, sandy loam
**Warwick**	Agriculture	N 52°19'01" W 1°31'19"	285	River terrace sand/gravel, glacial till	Sand, sandy loam, clayey loam
**Warwick**	Agriculture	N 52°19'21" W 1°26'19"	245	River terrace sand/gravel	Sandy loam
**Warwick**	Woodland	N 52°16'49" W 1°34'42"	164	Riverine clay, sands and gravels	Clay, sandy loam
**Warwick**	Woodland	N 52°17'30" W 1°30'38"	260	Claystone/mudstone	Clayey loam, silty loam
**Warwick**	Woodland	N 52°17'17" W 1°30'04"	210	River terrace sand/gravel	Sandy loam
**Warwick**	Grassland	N 52°16'55" W 1°34'22"	160	Riverine clay, sands and gravels	Clay, sandy loam
**Warwick**	Grassland	N 52°17'29" W 1°30'34"	275	Claystone/mudstone	Clayey loam, silty loam
**Warwick**	Grassland	N 52°17'18" W 1°30'10"	200	River terrace sand/gravel	Sandy loam
**Warwick**	Built	N 52°16'46" W 1°34'30"	160	River terrace sand/gravel	Sandy loam
**Warwick**	Built	N 52°17'30" W 1°31'02"	210	Claystone/mudstone	Clayey loam, silty loam
**Warwick**	Built	N 52°17'23" W 1°30'38"	200	Claystone/mudstone	Clayey loam, silty loam
**Coventry**	Agriculture	N 52° 26' 46" W 1°26' 17"	460	Glacial till	Clayey loam, sandy loam
**Coventry**	Agriculture	N 52°26'41" W1°26'24"	465	Glacial till	Clayey loam, sandy loam
**Coventry**	Agriculture	N 52° 26' 39" W 1°26' 5"	480	Glacial till	Clayey loam, sandy loam
**Coventry**	Woodland	N 52° 23' 14" W - 1° 32' 29"	290	Riverine clay, sands and gravels	Clay, sandy loam
**Coventry**	Woodland	N 52° 24' 41" W - 1° 32' 26"	480	Riverine clay, sands and gravels	Clay, sandy loam
**Coventry**	Woodland	N 52° 23' 27" W -1° 31' 16"	470	Mudstone and sandstone	Clayey loam, sandy loam
**Coventry**	Grassland	N 52° 23' 24" W - 1° 32' 26"	295	Mudstone and sandstone	Clayey loam, sandy loam

**Coventry**	Grassland	N 52° 24' 39" W - 1° 32' 9"	430	Riverine clay, sands and gravels	Clay, sandy loam
**Coventry**	Grassland	N 52° 23' 27" W - 1° 31' 16"	500	Sandstone	Loam, Sandy Loam
**Coventry**	Built	N 52° 22' 60" W - 1° 32' 35"	280	Mudstone and sandstone	Clayey loam, sandy loam
**Coventry**	Built	N 52° 24' 35" W - 1° 32' 7"	300	Sandstone	Loam, Sandy Loam
**Coventry**	Built	N 52° 23' 29" W - 1° 31' 22"	485	Mudstone and sandstone	Clayey loam, sandy loam

Table S2. Meteorological Details. Details of precipitation (mm) and temperature (°C) at the time of sampling (November 2021, February 2022, May 2022, August 2022). Data source: Met Office climate summaries, Met Office National Climate Information Centre HadUK-Grid 1km gridded climate data from land surface network.

Month	Mean UK Precipitation (mm)	Mean UK Temperature (°C)
**November 2021**	74.9	7.0
**February 2022**	146.2	5.6
**May 2022**	75.7	11.8
**August 2022**	50.8	16.7

Table S3. Physicochemical Analyses Significance Values. *P*-values obtained from Kruskal-Wallis rank sum tests. Where pH and moisture content (Method S2) were compared between locations (‘Low Population, Wellesbourne, UK; <1K’, ‘Mid population, Warwick, UK; 35-52K’, and ‘High Population, Coventry, UK; >400K’), land-use types (agricultural, woodland, grass-dominated and built), and seasons (spring, winter, summer). NS indicates non-significant results.

	Season	Location	Land-use
pH	NS	< 0.001	< 0.001
Moisture Content	< 0.001	NS	< 0.001

Table S4. Mean Values and Standard Error of Flux Measurements

	Season			Land-use Type				Location		
	Spring	Winter	Summer	Agriculture	Woodland	Grassland	Built	Low Population (Wellesbourne, UK; 1K)	Mid Population (Warwick, UK; 35-52K)	High Population (Coventry, UK; >400K)
F_{NO}	430.03 ±111.17	633.65 ±434.17	533.50 ±202.70	347.72 ±138.04	1234.88 ±657.48	305.50 ±120.33	339.76 ±120.46	556.36 ±189.06	291.47 ±100.44	705.34 ±423.88
$F_{NO2+NOZ}$	-54.80 ±66.12	-138.56 ±59.05	-245.16 ±202.84	-86.33 ±75.87	-414.52 ±253.21	-46.90 ±70.55	-62.96 ±38.75	-280.12 ±172.59	-113.68 ±35.56	-20.82 ±48.61
F_{NOY}	372.15 ±153.91	495.07 ±449.15	222.09 ±173.16	261.29 ±210.62	735.26 ±725.55	258.61 ±105.88	273.03 ±145.83	221.99 ±199.32	177.83 ±124.10	681.44 ±428.58

Table S5. ICP-OES standards used to obtain a calibration curve for ICP-OES analysis. Multi-element standard solution contained 23 elements in diluted nitric acid with a starting concentration of 1000 mg/L: Ag, Al, B, Ba, Bi, Ca, Cd, Co, Cr, Cu, Fe, Ga, In, K, Li, Mg, Mn, Na, Ni, Pb, Sr, Ti, Zn.

	mg/L
Blank	0
Calibration 1	0.1
Calibration 2	0.5
Calibration 3	1
Calibration 4	10
Calibration 5	50
Calibration 6	100

Table S6. Quantitative PCR Primers. Table of specific primers used for quantitative qPCR

**Gene**	**Function**	**Sequence**	**Optimal Annealing temp. (°C)**	**Source**
Commamox amoA	Complete ammonia oxidation (nitrification)	F: GGRACYTTYCAYATGCACACVGC<br>R: ACCACCAMGAVGTRTARACSGCAA	50	Keeley *et al.*, 2020
Periplasmic nxrB	Nitrite oxidoreductase (nitrification)	F: GTGGAACAAYGTGGARACSAAGCC<br>R: SACRAASCGCCAYTCYTGGTC	55	Keeley *et al.*, 2020
Bacterial amoA	Ammonia oxidation to nitrite (nitrification)	F: GACTGGGAYTTCTGGMTKGAYTGGAA<br>R: TGYGACCACCAGTARAAWCCCCAG	55	Keeley *et al.*, 2020
Archaeal amoA	Ammonia oxidation to nitrite (nitrification)	F: GCMTTCAARTATCCRAGRCCRACRYTGCCW<br>R: TACMGATGGATGGCCGCNTGGWSMAAG	55	Francis *et al.*, 2005

Figures

Figure S1. Map of Sampling Sites

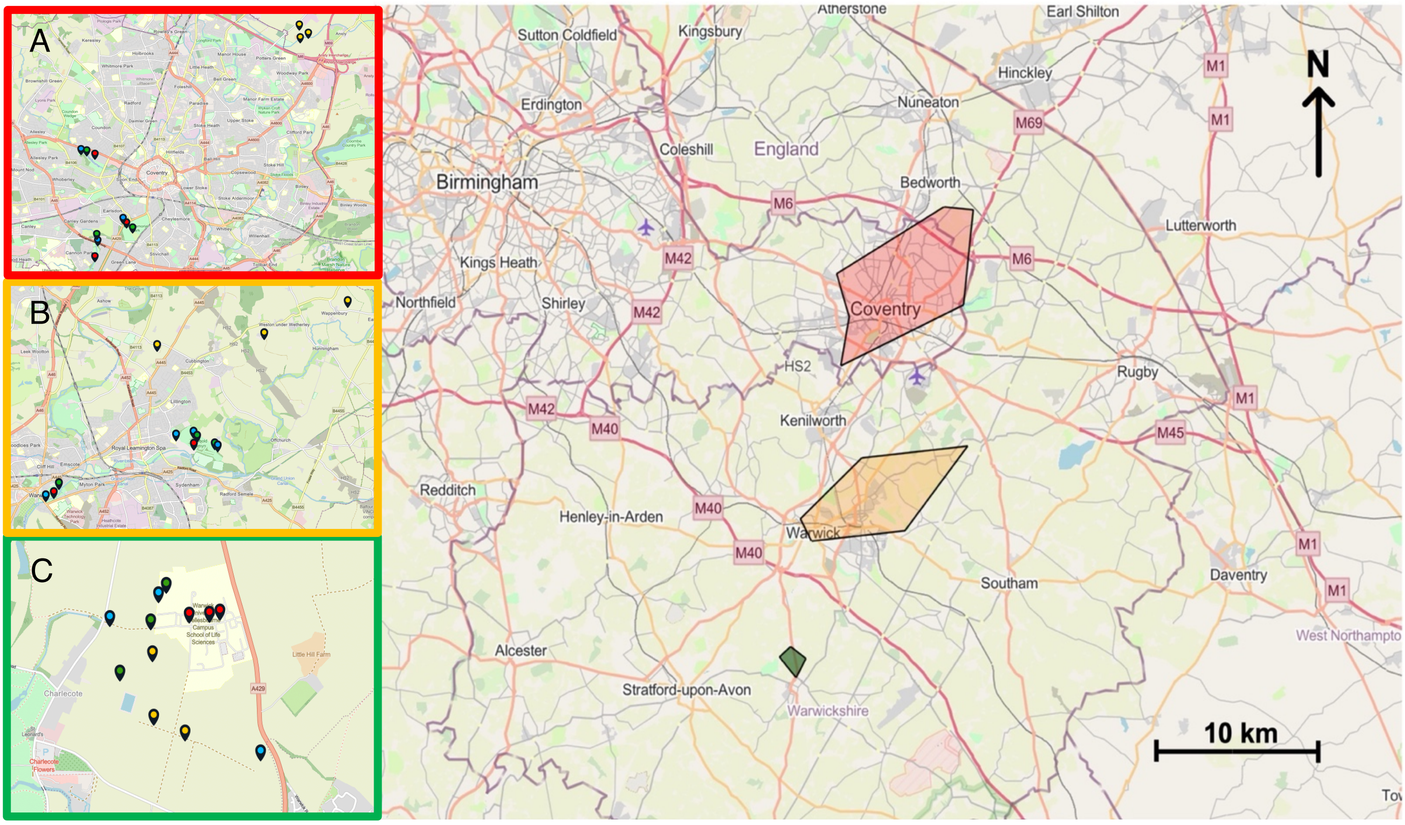

Figure S1. Map showing sampling sites. A) High Population, Coventry, UK; >400K. B) Mid population, Warwick, UK; 35-52K. C) Low Population, Wellesbourne, UK; <1K. Yellow markers indicated "agriculture" samples, blue markers indicate "woodland" samples, green markers indicate "grass-dominated" samples, red markers indicate "built" samples.

Figure S2. Soil Physicochemical Properties

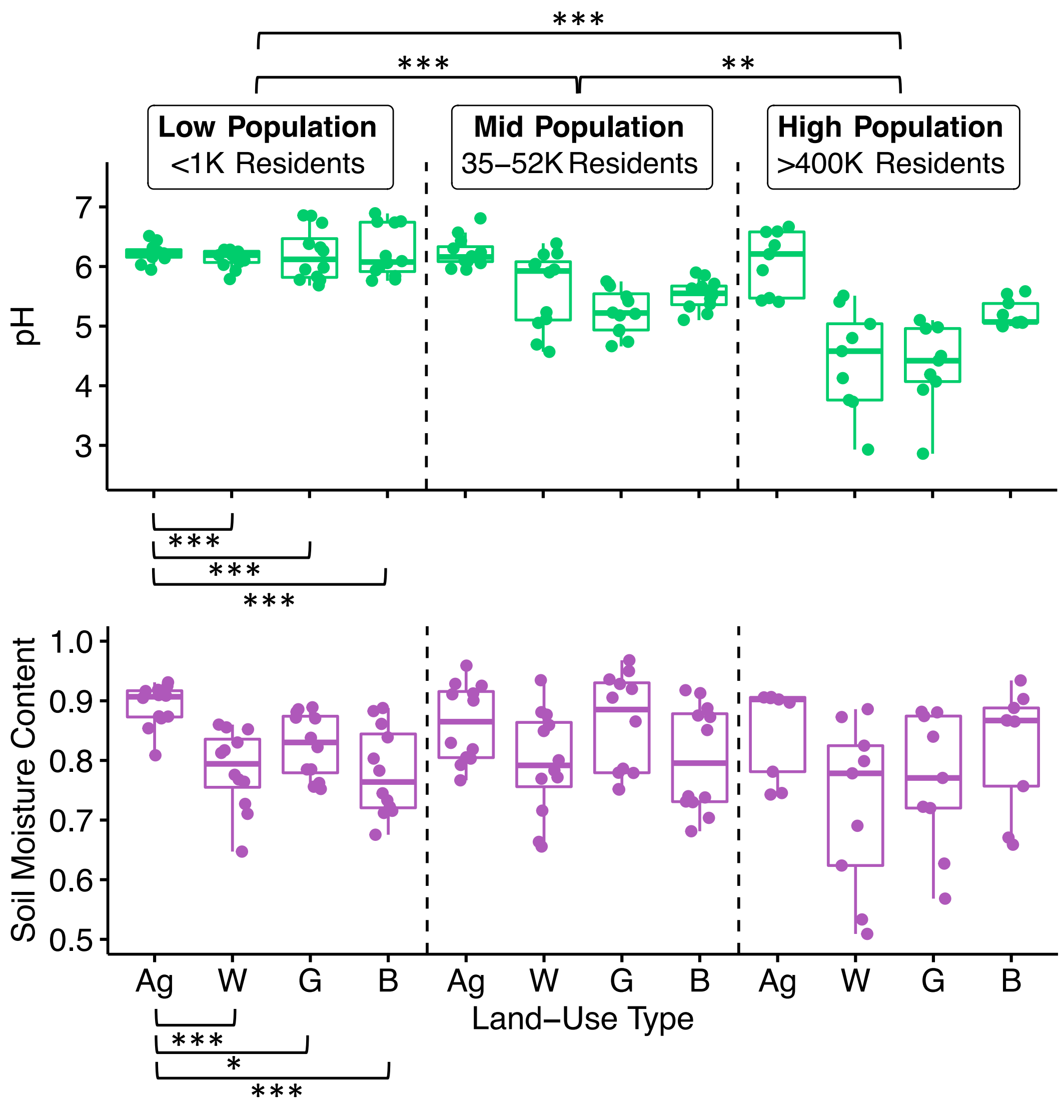

Figure S2. pH and moisture content of soil samples taken from three locations: 'Low Population, Wellesbourne, UK; <1K', 'Mid population, Warwick, UK; 35-52K', and 'High Population, Coventry, UK; >400K', and across four land-use types: 'Ag' = Agriculture; 'W' = Woodland; 'G' = Grass-dominated; 'B' = Built (defined as soil taken from < 1m from a human-made structure). Significance lines indicate significant differences in pH and moisture content between locations and land-use types determined by Kruskal-Wallis rank sum tests (* = < 0.05, ** = < 0.01, *** = < 0.001). $N = 132$.

Figure S3. Available NO_3^- and NH_4^+ in Agricultural Soils

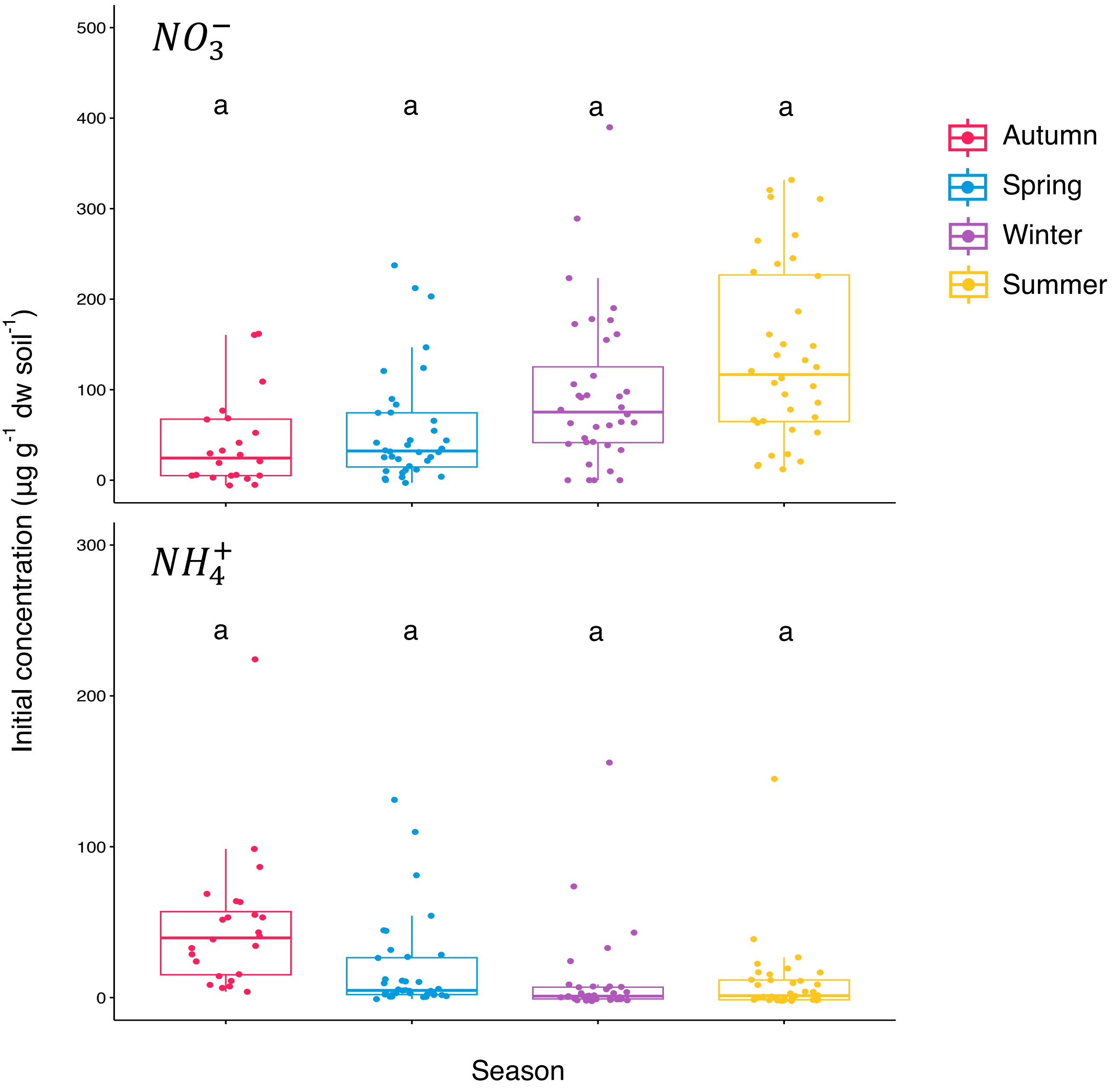

Figure S3. Effect of season on available NO_3^- and NH_4^+ in agricultural soil samples. Concentrations of NO_3^- and NH_4^+ were quantified using a segmented flow analyser. Letters indicate significant differences in NO_3^- and NH_4^+ between seasons (autumn, spring, winter, summer) determined by Kruskal-Wallis rank sum tests. Variables with the same letter are not significantly difference. Variables with different letters are significantly different. N = 33.

Figure S4. NOy Flux Analysis Setup

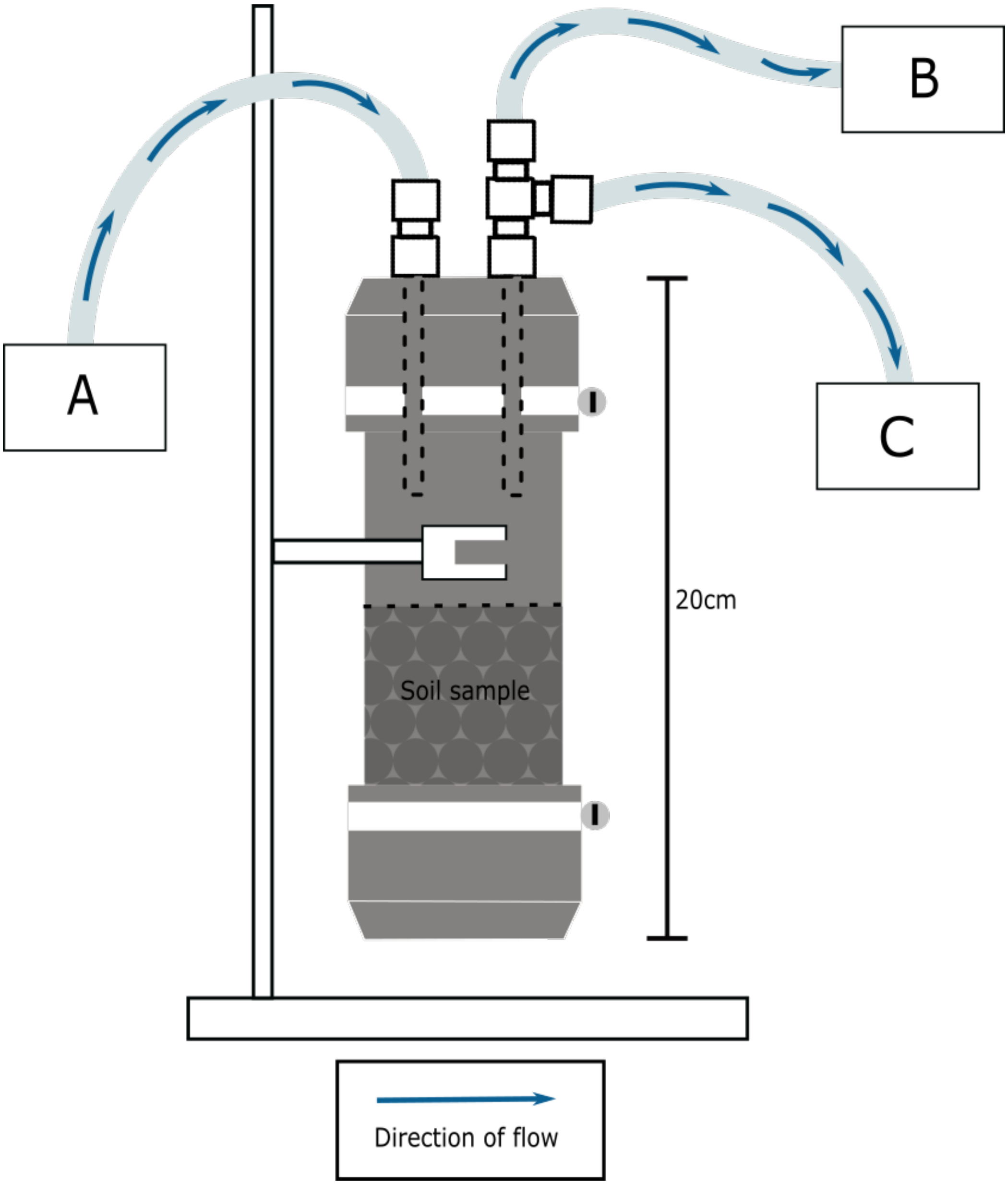

Figure S4. System for measuring NOy gas fluxes from intact soil cores. A. Pure air flows into the system from a zero-air source (flow rate = 2.0 L min^{-1}) through a one-way PTFE union. B. To calculate flux, flow rate at the start and end of the sampling period is measured using a masterflex digital flow meter. By blocking port C, we measure flow out of the system, which on average was 1.7 L min^{-1}. C. NOy is quantified by a Teledyne T200U-NOy analyser by sampling off the excess flow line at a rate of 0.9 L min^{-1}.

Figure S5. Potential Mean NOy Fluxes

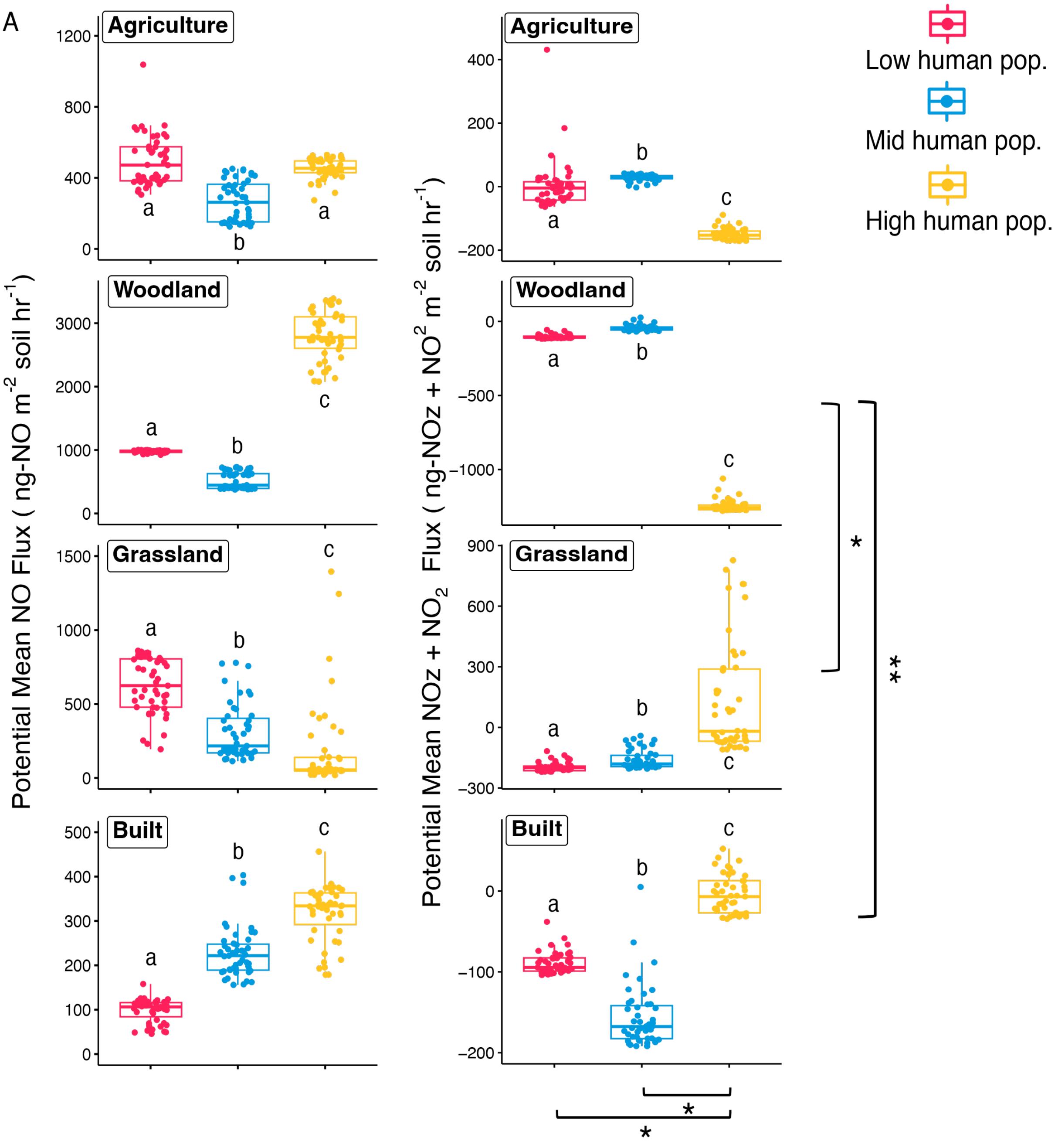

Figure S5. The effect of land-use type (agricultural, woodland, grass-dominated, built) and location ('Low Population, Wellesbourne, UK; <1K', 'Mid population, Warwick, UK; 35-52K', and 'High Population, Coventry, UK; >400K') on potential mean fluxes of NO (F_{NO}) and NO_2 + NOz ($F_{NO2+NOZ}$). Fluxes were measured with a chemiluminescence technique using a Teledyne T200U instrument. N = 36. Significance lines indicate significant differences in fluxes between seasons (* = < 0.05, ** = < 0.01, *** = < 0.001).

Figure S6. Annual Nitrogen Deposition.

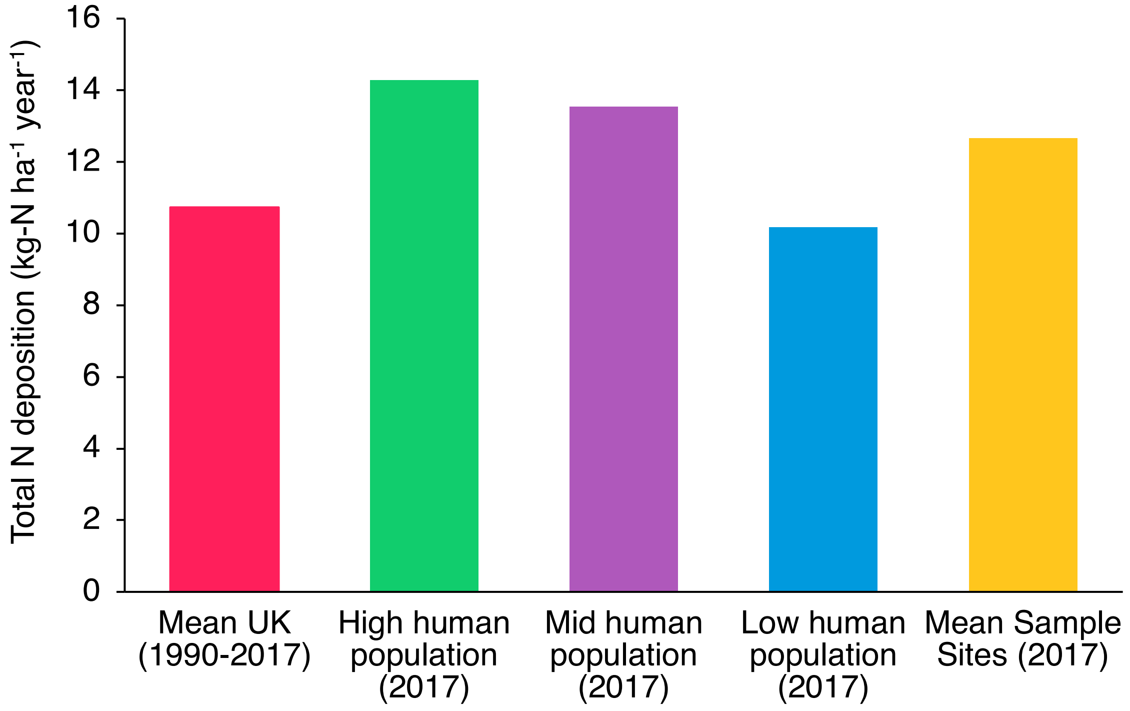

Figure S6. Annual nitrogen (N) deposition in the UK at 1 km resolution. N deposition was calculated as wet reduced N (NHx) + dry NHx + wet oxidised N (NOy) + dry NOy deposition. Data is presented for the mean UK N deposition (1990-2017), high human population sample site (Coventry, UK, 2017), mid human population sample site (Warwick, UK, 2017), low human population site (Wellesbourne, UK, 2017), and mean of all sample sites (2017). Data source: Tomlinson, S.J.; Carnell, E.J.; Dore, A.J.; Dragosits, U. Nitrogen deposition in the UK at 1km resolution, 1990-2017**.** *NERC Environmental Information Data Centre. (Dataset),* **2020**; doi: 10.5285/9b203324-6b37-4e91-b028-e073b197fb9f.

Figure S7. Relative Abundance of Total Microbial Community

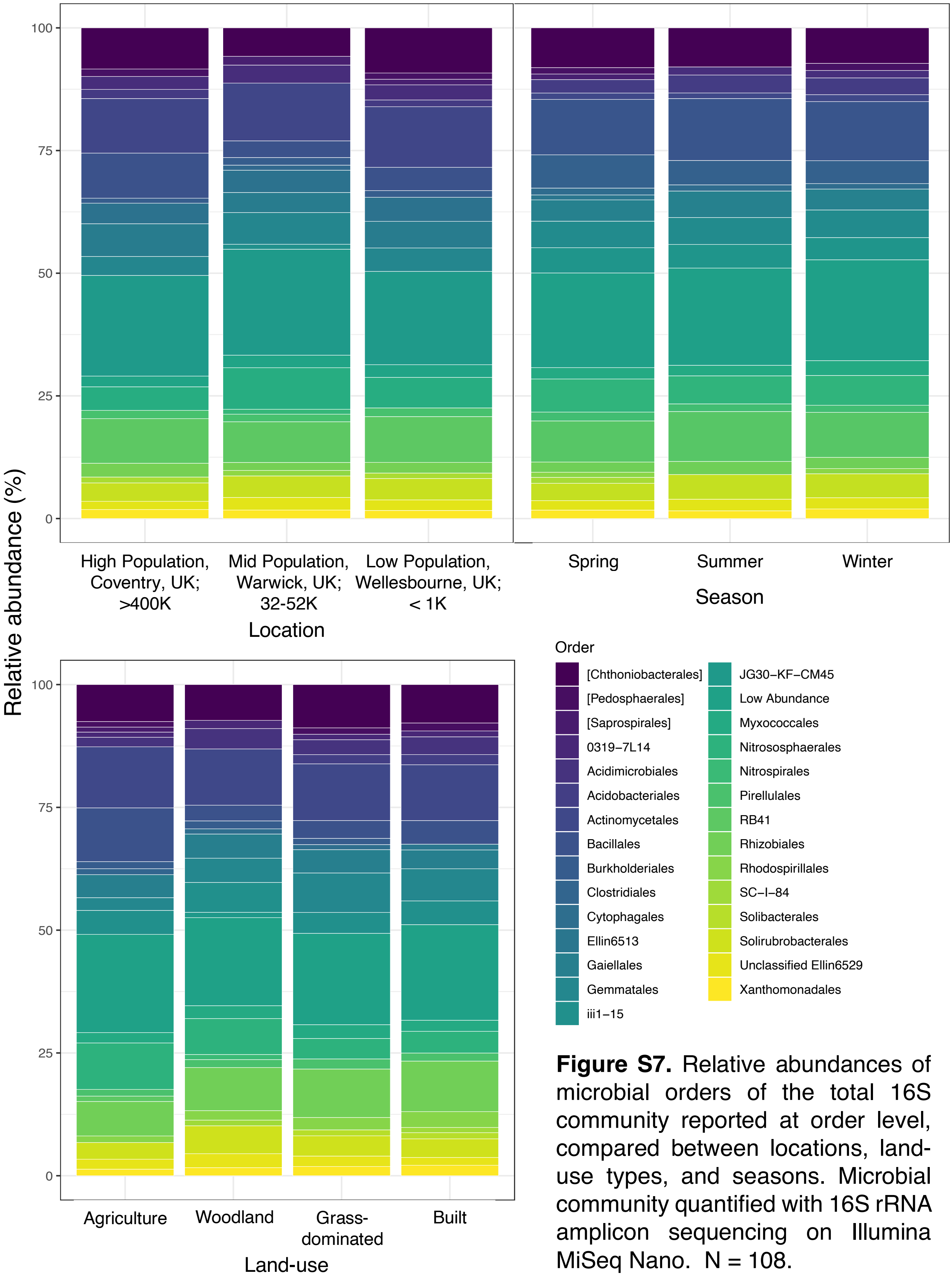

Figure S7. Relative abundances of microbial orders of the total 16S community reported at order level, compared between locations, land-use types, and seasons. Microbial community quantified with 16S rRNA amplicon sequencing on Illumina MiSeq Nano. N = 108.

Figure S8. Fisher's Alpha Diversity Index

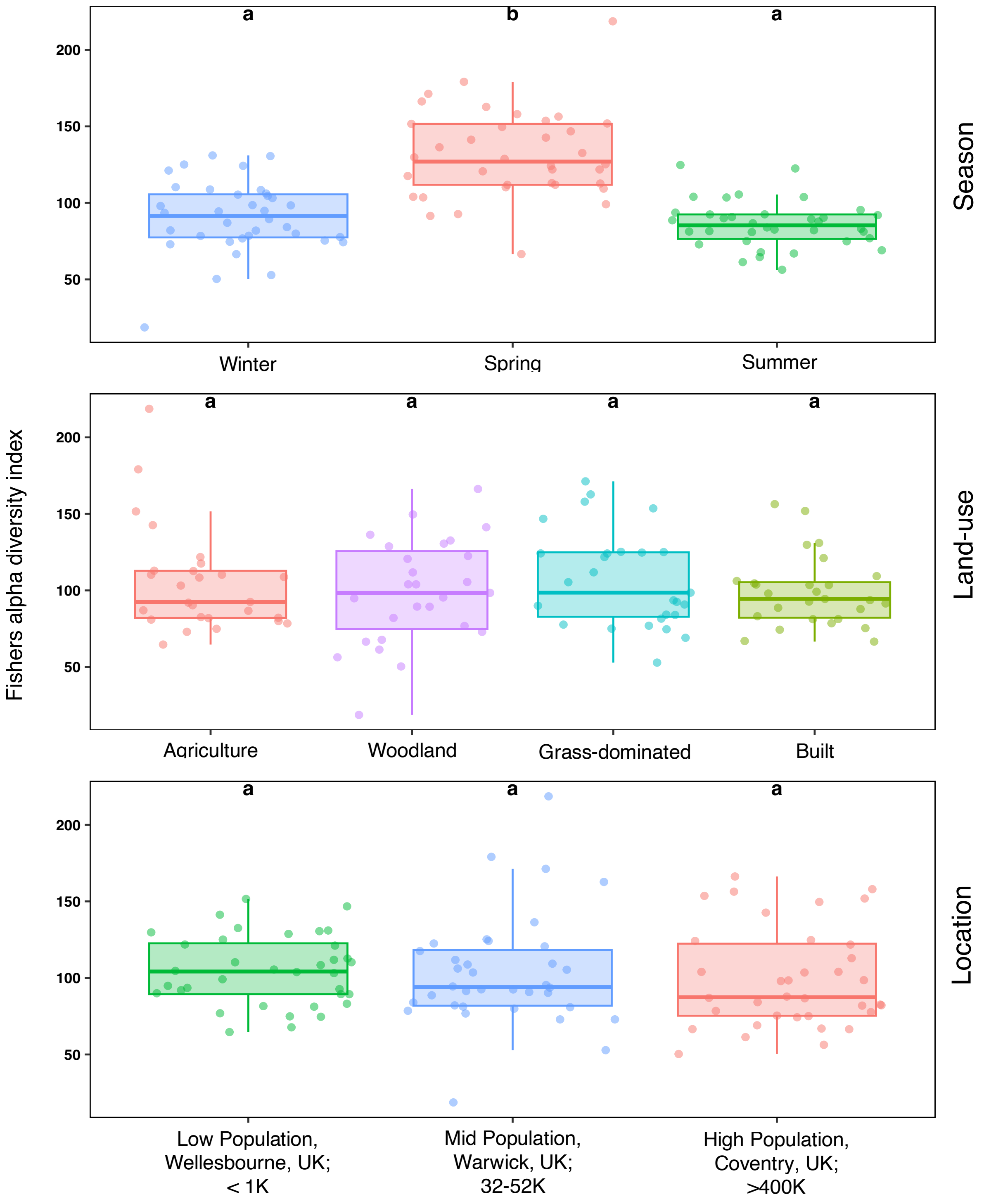

Figure S8. Fishers alpha diversity index analysis. Letters indicate significant differences in diversity between locations ('Low Population, Wellesbourne, UK; <1K', 'Mid population, Warwick, UK; 35-52K', and 'High Population, Coventry, UK; >400K'), land-use types (agricultural, woodland, grass-dominated and built), and seasons (spring, winter, summer). Variables with the same letter are not significantly difference. Variables with different letters are significantly different. N = 108.

Figure S9. Non-metric Multidimensional Scaling Analysis

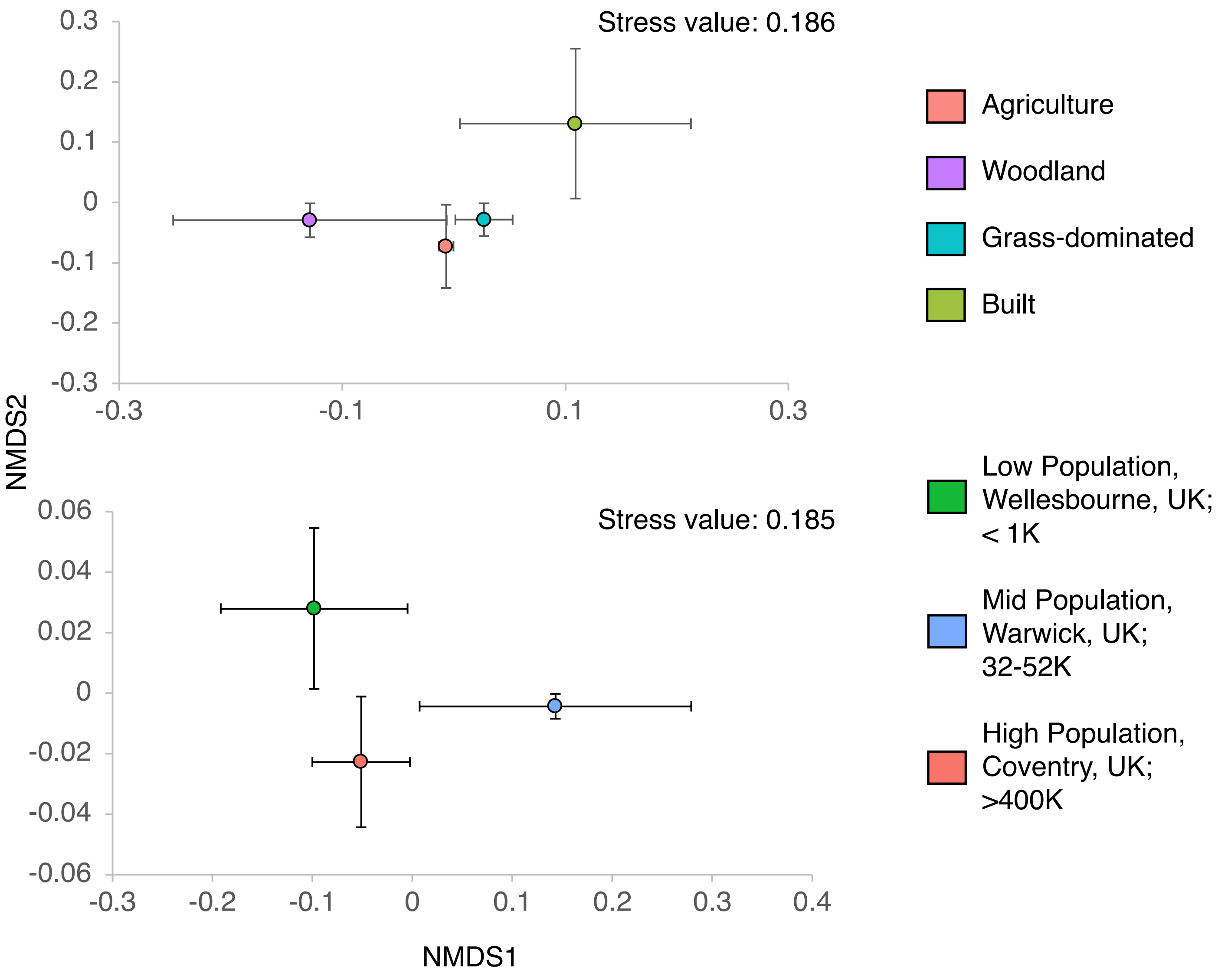

Figure S9. Values from Non-metric Multidimensional Scaling (NMDS) analysis averaged over land-use types (agricultural, woodland, grass-dominated, built) and locations ('Low Population, Wellesbourne, UK; <1K', 'Mid population, Warwick, UK; 35-52K', and 'High Population, Coventry, UK; >400K') with 95% confidence error bars. N = 108.

Figure S10. Heavy Metal Concentrations

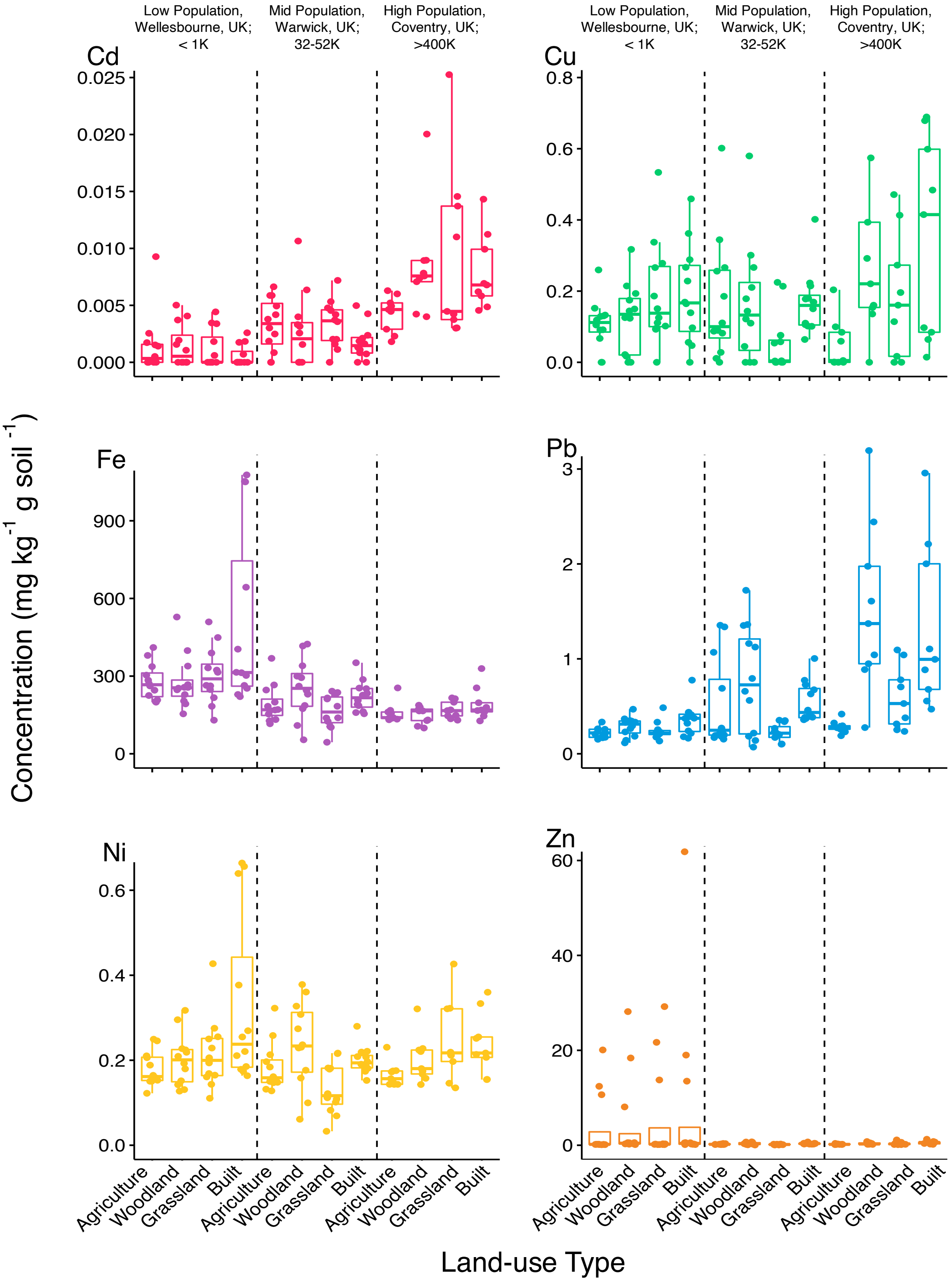

Figure S10. Concentrations of heavy metals (Cd, Cu, Fe, Pb, Ni, Zn) measured using ICP-OES across land-use types (agricultural, woodland, grass-dominated, built) and locations ('Low Population, Wellesbourne, UK; <1K', 'Mid population, Warwick, UK; 35-52K', and 'High Population, Coventry, UK; >400K'). Significance lines indicate results from Kruskal Wallis with Dunn's tests where heavy metal concentrations were compared between locations and land-use types (∗ = < 0.05, ∗∗ = < 0.01, *** = < 0.001). N = 108.

Figure S11. Effect of Road Proximity on Heavy Metal Concentrations

A

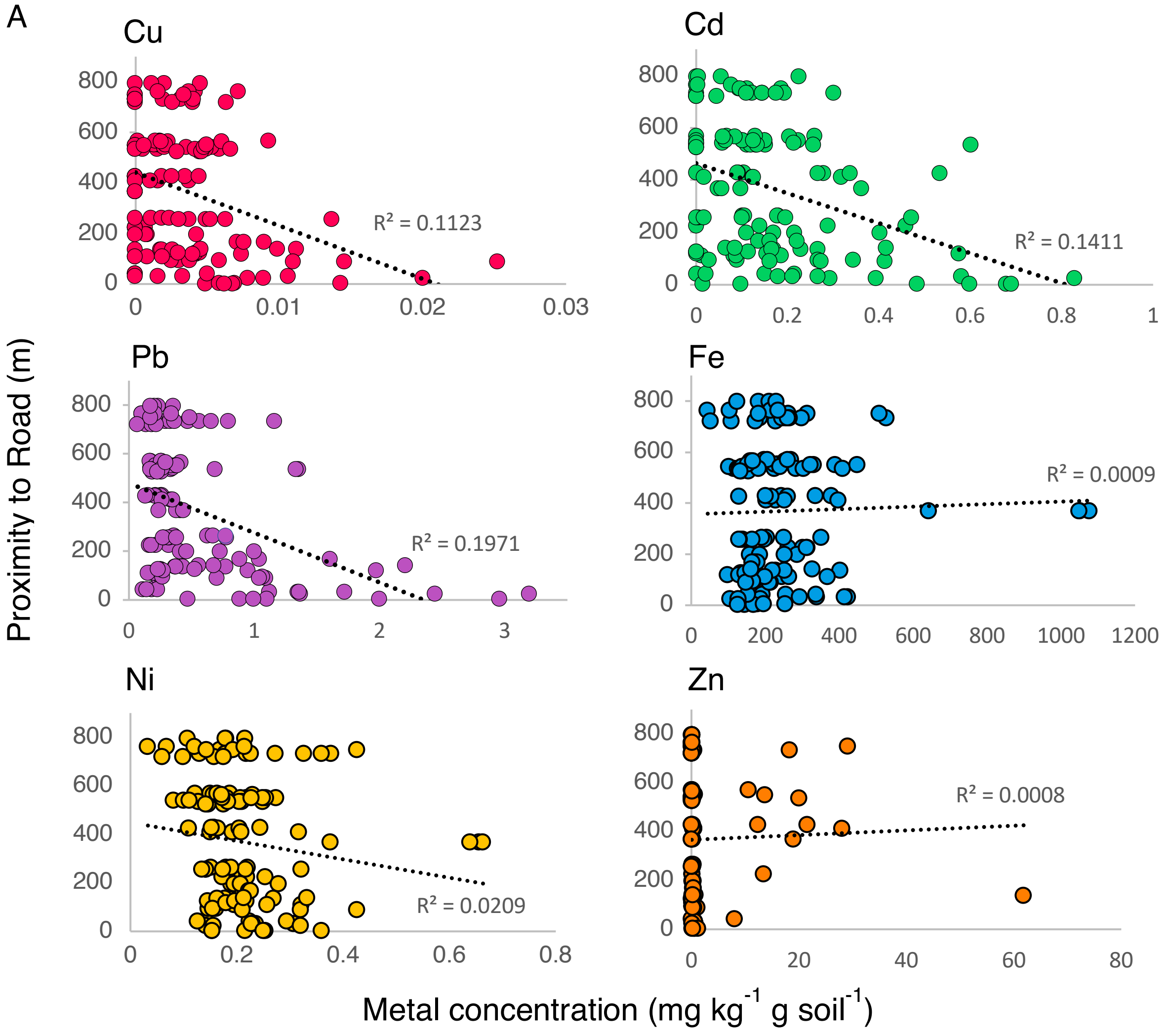

B

	Season	Location	Land-use
Cd	NS	NS	< 0.001
Cu	NS	< 0.05	NS
Fe	NS	NS	< 0.001
Pb	NS	< 0.001	< 0.001
Ni	NS	< 0.01	NS
Zn	< 0.05	< 0.001	NS

Figure S11. A. Concentrations of heavy metals (Cd, Cu, Fe, Pb, Ni, Zn) measured using ICP-OES plotted against proximity of sample sites to the closest roads. **B.** *P*-values obtained from Kruskal-Wallis rank sum tests where concentrations of heavy metals were compared between locations (‘Low Population, Wellesbourne, UK; <1K’, ‘Mid population, Warwick, UK; 35-52K’, and ‘High Population, Coventry, UK; >400K’), land-use types (agricultural, woodland, grass-dominated and built), and seasons (autumn, spring, winter, summer). NS indicates non-significant results. N = 132.

Figure S12. Simulated N Deposition Continuous Flux Measurements

A

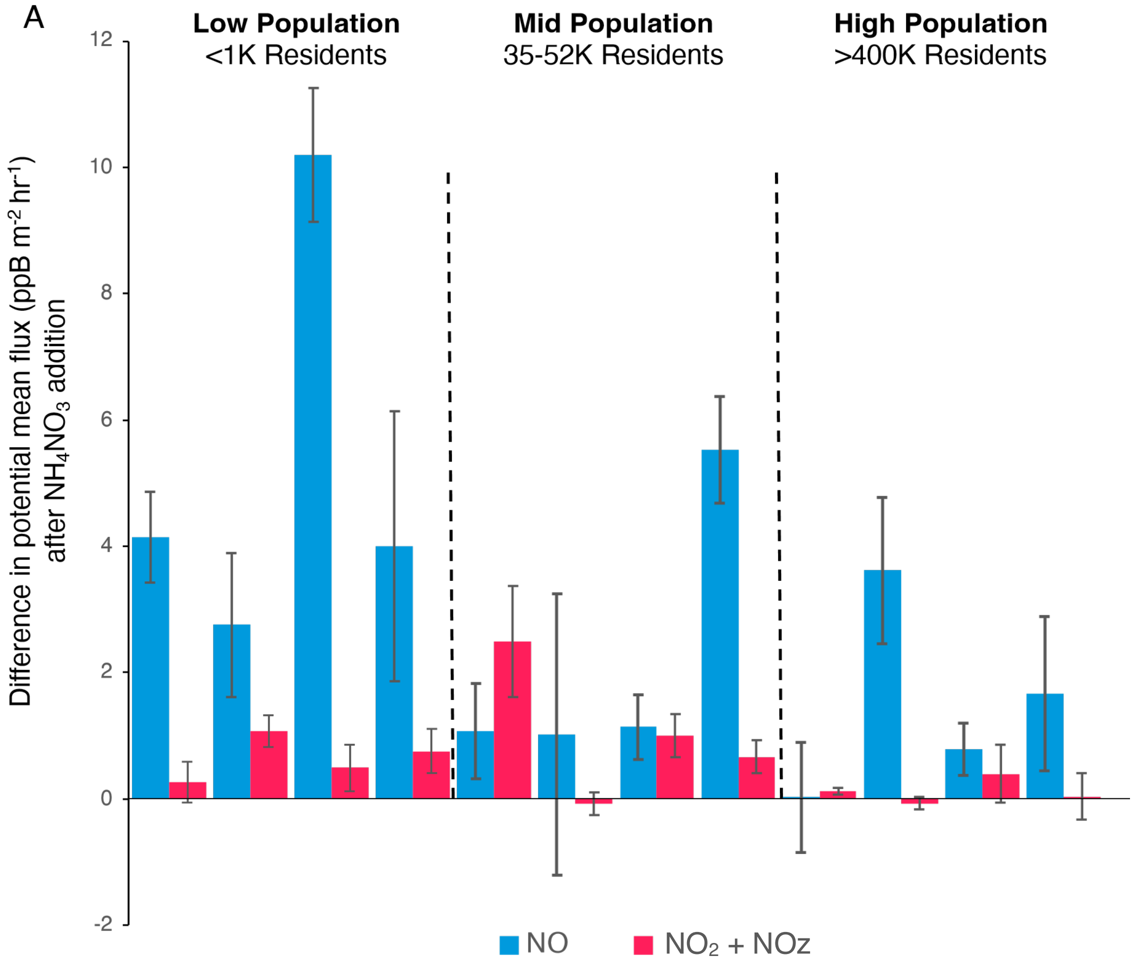

B

	Season	Location	Land-use
Difference in F_{NOY}	NS	NS	NS
Difference in F_{NO}	NS	< 0.05	NS
Difference in $F_{NO2+NOZ}$	NS	NS	NS

Figure S12. A. Difference in potential mean fluxes of NO and NO_2 + NOz, measured with a chemiluminescence technique using a Teledyne T200U instrument, before and after NH_4NO_3 addition to simulate 4 months nitrogen (N) depositions. N = 36 **B**. Table of p-values obtained from Kruskal-Wallis rank sum tests where differences in F_{NOY}, F_{NO} and $F_{NO2+NOZ}$ before and after simulated N deposition were compared between locations ('Low Population, Wellesbourne, UK; <1K', 'Mid population, Warwick, UK; 35 -52K', and 'High Population, Coventry, UK; >400K'), land-use types (agricultural, woodland, grass-dominated and built), and seasons (spring, winter, summer). NS indicates non-significant results.

Figure S13. Structural Equation Modelling R Values

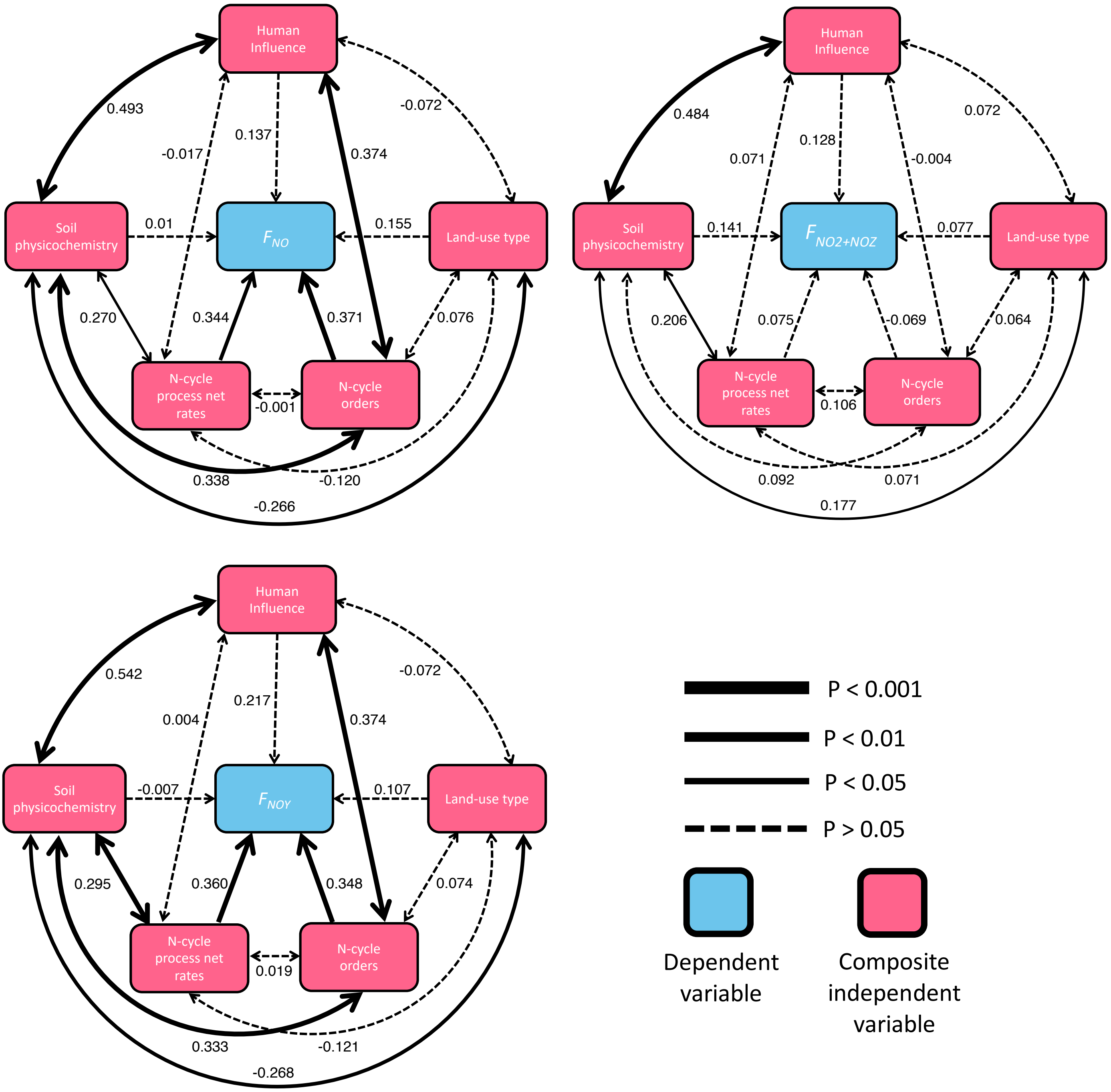

Figure S13. Structural equation modelling to ascertain effects of measured variable on fluxes of NO (F_{NO}), NO_2 + NOz ($F_{NO2+NOZ}$), and NOy (F_{NOY}). Fixed effects: Composite soil physicochemical properties (pH and moisture content, N = 132); composite relative abundances of nitrogen (N) cycle associated microbial taxa, N = 108; composite net rates of N cycle processes (nitrification, ammonification, N mineralisation), N = 132; land-use type (agricultural, woodland, grass-dominated and built, N = 132), and human influence, N =132. Season (autumn, spring, winter, summer) is a random effect. The "human influence" variable is the result of a factorial analysis of mixed data (FAMD) to condense heavy metal concentrations from ICP-OES analysis, road proximity, and sample location ('Low Population, Wellesbourne, UK; <1K', 'Mid population, Warwick, UK; 35-52K', and 'High Population, Coventry, UK; >400K') into single values. Line labels are correlation coefficient (R) values. N = 132
